# Single Pathogen Challenge with Agents of the Bovine Respiratory Disease Complex

**DOI:** 10.1371/journal.pone.0142479

**Published:** 2015-11-16

**Authors:** Laurel J. Gershwin, Alison L. Van Eenennaam, Mark L. Anderson, Heather A. McEligot, Matt X. Shao, Rachel Toaff-Rosenstein, Jeremy F. Taylor, Holly L. Neibergs, James Womack, Bovine Respiratory Disease Complex Coordinated Agricultural Project Research Team

**Affiliations:** 1 Department of Pathology, Microbiology, & Immunology, School of Veterinary Medicine, University of California, Davis, California, United States of America; 2 Department of Animal Science, College of Agriculture, University of California, Davis, California, United States of America; 3 Division of Animal Sciences, University of Missouri, Columbia, Missouri, United States of America; 4 Department of Animal Sciences, College of Veterinary Medicine, Washington State University, Pullman, Washington, United States of America; 5 Department of Veterinary Pathobiology, Texas A & M University, College Station, Texas, 77843–4467, United States of America; University of Alabama at Birmingham, UNITED STATES

## Abstract

Bovine respiratory disease complex (BRDC) is an important cause of mortality and morbidity in cattle; costing the dairy and beef industries millions of dollars annually, despite the use of vaccines and antibiotics. BRDC is caused by one or more of several viruses (bovine respiratory syncytial virus, bovine herpes type 1 also known as infectious bovine rhinotracheitis, and bovine viral diarrhea virus), which predispose animals to infection with one or more bacteria. These include: *Pasteurella multocida*, *Mannheimia haemolytica*, *Mycoplasma bovis*, and *Histophilus somni*. Some cattle appear to be more resistant to BRDC than others. We hypothesize that appropriate immune responses to these pathogens are subject to genetic control. To determine which genes are involved in the immune response to each of these pathogens it was first necessary to experimentally induce infection separately with each pathogen to document clinical and pathological responses in animals from which tissues were harvested for subsequent RNA sequencing. Herein these infections and animal responses are described.

## Introduction

The bovine respiratory disease complex (BRDC) is a multi-agent disease of cattle often referred to as “shipping fever.” It has been and continues to be one of the largest causes of morbidity and mortality of dairy calves and feedlot cattle [1]. Despite rigorous research and development of vaccines containing some or all of the major BRDC pathogens, the disease remains a major cause of financial loss to the cattle industry. BRDC is associated with several bacterial pathogens, of which the major ones are: *Pasteurella multocida*, *Mannheimia haemolytica*, *Mycoplasma bovis*, and *Histophilus somni*. Disease occurs when a viral pathogen: Bovine respiratory syncytial virus (BRSV), Bovine Herpes virus type 1 (infectious bovine rhinotracheitis, IBR), Bovine parainfluenza 3 virus, or Bovine viral diarrhea virus (BVDV) infects the host and weakens resistance to the bacterial pathogen, which is often already present in the upper respiratory tract. Stress is another factor that facilitates the development of BRDC. The term “shipping fever” is often used because the stress of shipping cattle to a feed yard facilitates the suppression of immune responses at a time when animals are mixed together in close quarters allowing viral transmission [2].

Each of the BRD pathogens has unique features that contribute to the ability of the pathogen to cause disease and to resist host defenses. These are summarized in Table 1 for the pathogens used in the infection studies described herein.

**Table 1:** Characteristic Features of Major BRD Pathogens.

**Viral Pathogens**			
**Pathogen**	**Characteristics**	**Mechanisms of Pathogenesis**	**Effect on Host Response**
Bovine Respiratory Syncytial Virus	Negative strand RNA virus, Paramyxoviridae	Entry through respiratory mucosa; infects bronchial epithelium, causes syncytial cell formation, bronchiolitis. Fever, cough, increased respiratory rate, depression	Immune modulation favoring T helper type 2 cytokines, which depresses cytotoxic T cell induction [15]
Bovine Herpes Virus 1 (IBR)	DNA virus, Herpesviridae, alphavirinae	Entry through respiratory mucosa; causes epithelial cell apoptosis. Fever, rhinotracheitis, cough, conjunctivitis, oral ulcers; reproductive tract infection with abortion^5^	Evades host defenses: depresses Interferon 1 responses, latency, suppresses CMI by interfering with TAP peptide MHC 1, interference with chemokine function [16,17]
Bovine Viral Diarrhea Virus	Positive strand RNA virus, Flaviviridae, two biotypes 1 and 2	Spread in secretions; causes multiple system disease (abortion, persistent infection.	Causes immunosuppression, targeting and killing lymphoid tissue in Peyer’s Patches [12,18]
**Bacterial Pathogens**			
*Pasteurella multocida*	Gram negative coccobacillus, family Pasteurellaceae	Part of normal microbiota in upper respiratory tract; stress or viral infections allow it to infect lung and cause bronchopneumonia	Multiple virulence factors: anti-phagocytic capsule, LPS, protein toxin[11,13,19]
*Mannheimnia hemolytica*	Gram negative coccobacillus, family Pasteurellaceae	Upper respiratory tract commensal; opportunistic pathogen causing bronchopneumonia	Multiple virulence factors: adhesin, capsular poly-saccharide, leukotoxin, LPS, transferrin binding protein [20,21]
*Mycoplasma bovis*	Wall-less bacterium of class Mollicutes	Causes mastitis, arthritis, otitis media, pneumonia, fever, cough, anorexia, nasal discharge; synergistic with other BRD pathogens, forms biofilms to facilitate persistence	Variable surface membrane lipoprotein antigens, adhesins, immune modulation (to Th2), inhibits neutrophil respiratory burst [22,23]
*Histophilus somni*	Gran negative coccobacillus, Pasteurellaceae	Upper respiratory tract, reproductive tract commensal; Diseases: thrombotic meningoencephalitis (TME), respiratory disease, myocarditis, polysynovitis, otitis media, mastitis, and reproductive tract diseases.	Virulence factors: LPS, LOS, apoptosis of endothelial cells,Immunoglobulin binding protein [24].

The aim of this study was to develop and characterize experimental infection protocols for each of the BRC pathogens. We hypothesize that each of these organisms elicits a particular immune response from the host and that the most appropriate response can be identified by evaluation of gene usage in single pathogen infections. To determine which genes are involved in a protective immune response it is necessary to experimentally induce each infection separately and to monitor clinical and pathological responses in infected animals using these infection protocols. Necropsy tissues can be harvested to serve as a source of RNA for sequence analysis and cells can be obtained for analysis of the immune response [3].

## Methods

### Animals and husbandry

Angus-sired crossbred steers were obtained from the Sierra Field Station of the University of California Davis (UCD) located in Brown’s Valley, CA where they had been raised since birth. This study was carried out in strict accordance with the recommendations in the Guide for the Care and Use of Laboratory Animals of the National Institutes of Health. The protocol (#16424) was approved by the University of California, Davis, Institutional Animal Care and Use Committee. Blood was obtained and titers were determined against each viral pathogen, and those seronegative or with lowest titers were chosen for use in the experiment. Six to eight month old steers were shipped to UCD where they were housed in pens of 4 each at the University’s Animal Science cattle facility. All facilities were thoroughly disinfected between pathogen challenges. The steers were fed a starter diet consisting of 65% concentrate and water *ad libitum*. A total of six steers were used for each pathogen challenge (2 in the pilot study and 4 in the optimal study) and a total of three steers were used for viral controls and an additional three for the bacterial challenge controls. The Angus-Hereford cross steers were used partially due to availability and more importantly to have a broader genetic composition included in the resultant tissue bank than would result from using purebred cattle in line with the ultimate goal of creating an RNA tissue source that is broadly representative of cattle. A power calculation performed to determine the appropriate number of animals to use for a 95% confidence interval was performed using our previous data on experimental BRSV infection. We used clinical scores in controls versus experimentally infected calves for the power analysis.

### Experiment Design

An initial pilot study was carried out in 2011 to determine the appropriate time course for each experimental infection so that tissue samples could be harvested at the time of maximal clinical signs (see Pilot Study results). The following year a larger challenge study utilizing additional animals per treatment for increased statistical significance was carried out using optimal conditions as informed by the pilot study. For some pathogens conditions and necropsy timing remained unchanged between the pilot and the challenge trial (IBR, BRSV). Whereas for the other pathogens different pathogen isolates and/or timing for infection was implemented based on the results from the pilot trial (Table 2). *Histophilus somni* was included in the pilot study, but did not achieve sufficient clinical signs or re-isolation of the bacterium to provide useful data. Animals available for the optimal study were inadvertently vaccinated for *H*. *somni*, making them unacceptable for use. Thus, *H*. *somni* data is not included herein. Each year infections were performed sequentially in groups of steers for each pathogen after a rest period to clean and disinfect facilities.

**Table 2:** Pathogens, doses, and animals.

Pathogen	Source	Dose Administered	Animal IDs
Bovine Respiratory Syncytial Virus	Dr. Laurel Gershwin—University of California, Davis (CA-1)	1.6 × 10^5^/ml ×8.5 ml/animal (both)	18, 33 (pilot^1^) 74, 77, 92, 116 (optimized^1^)
Bovine Herpes Virus– 2 (IBR)	CAHFS^2^ (ATCC, lot: VR188 LA strain)	1.0 × 10^7^/ml × 8.5 ml/animal (both)	81, 138 (pilot) 59, 70, 76, 100 (optimized)
Bovine Viral Diarrhea Virus	Dr. Chris Chase—South Dakota State University, (890)	2.4 × 10^7^/animal (pilot); 1.7 × 10^9^/ animal (optimized)	82, 128 (pilot) 86, 98, 138, 139 (optimized)
*Mannheimnia haemolytica*	CAHFS^2^ (pilot) Dr. Anthony Confer- Oklahoma State University (optimized)(232)	9.6 × 10^7^ CFU/animal (pilot) 4.8 x 10^11^ CFU/animal (optimized)	73, 75 (pilot) 88, 99, 113, 125 (optimized)
*Pasteurella multocida*	CAHFS^2^ (pilot) Dr. Anthony Confer- Oklahoma State University (optimized) (89020807N)	3.6 × 10^9^ CFU/animal (pilot) 1.13 x 10^11^ CFU/animal (optimized)	111, 129 pilot (pilot) 75, 123, 127, 135 (optimized)
*Mycoplasma bovis*	CAHFS^2^ (pilot) Dr. Ricardo Rosenbush- Iowa State University (optimized) (428E)	3.5 ×10^10^ CFU/animal (pilot) 7.0 × 10^10^ CFU/animal (optimized)	43, 124 (pilot) 47B, 65, 104^3^,115 (optimized)
Virus control (media)^4^	NA	Tissue culture media- same volume	25 (pilot); 58, 101 (optimized)
Bacterial control (PBS)^4^	NA	PBS—same volume	70 (pilot); 71, 102 (^optimized^)

NA = not applicable.

^1^The pilot study was conducted in summer 2011. The optimized study was conducted in summer 2012.

^2^California Animal Health and Food Safety Laboratory, clinical isolate.

^3^Euthanized early, tissues not sampled.

^4^For each pathogen in the optimized study there were 4 control steers for clinical sign analysis (a total of 24 steers); these were not necropsied. Tissue samples and pathology data was obtained from 3 additional numbered viral controls and 3 additional bacterial controls.

### Bacterial agents; methods of infection

The numbers of animals infected, source and dose of the virus, bacteria and mycoplasma strains, used for infection in the pilot (2011) and challenge (2012) studies are listed in Table 2. The total number of animals infected with each pathogen was six. A power calculation was performed for BRSV (the pathogen with which we had the greatest experience and data) and an n = 6 was determined to be sufficient for a 95% confidence interval. Data from pilot and optimal studies were combined to achieve an “n” of 6.

*Mannheimia haemolytica* was grown on blood heart infusion (BHI) agar with 5% sheep blood in a 5% CO_2_ incubator at 37°C for 24 hours. A growth curve was determined and correlated with optical density using isolated colonies grown at room temperature in BHI broth with shaking for 8 hours with sampling at 0–9 hours. Plate counts were performed to determine log phase growth. The growth curve was repeated 3 times to assure reproducibility, with 1 × 10^9^ CFU/ml corresponding to an OD_650_ of 0.809 after 7 hours of growth. Inoculum was prepared by growing the bacteria for 7 hours, concentrated by centrifugation followed by washing in cold phosphate buffered saline solution (PBS). Inoculum was prepared immediately prior to infection and held on wet ice until administered. In the optimized experiment a dose of 40 ml containing a total of 4.8 × 10^11^CFU/animal was administered to each steer intratracheally. The inoculum was followed by 20 ml of sterile PBS and 60 ml of air to assure that the entire inoculum was delivered into the respiratory tract.

*Pasteurella multocida* was grown on BHI as described for *M*. *haemolytica* and a growth curve was determined and correlated with an optical density value for log phase growth. In the optimized experiment each steer received by the intratracheal route 60 ml of bacteria for a total dose of 1.13 X 10^11^ CFU/animal, followed by 40 ml of cold sterile PBS and 60 ml of air.

*Mycoplasma bovis* was grown from a frozen seed stock culture for two days in PPLO broth in a 5% CO_2_ incubator at 37°C without shaking. A frozen stock was prepared and stored at -80°C. A small aliquot was titered; plate counts were performed and the titer of the stock was determined to be 3 × 10^10^ CFU/ml. On the day of infection, inoculum was prepared by diluting the frozen stock to 1 × 10^9^ CFU/ml. In the optimized experiment each steer received 7 × 10^10^ CFU by the intratracheal route followed by PBS and air as described above. Plate counts were set up to confirm the actual dose per steer. Bacterial infection controls (n = 3) received a similar inoculum of media (without bacteria) followed by PBS and air.

### Viral agents; methods of infection

Strains and amounts of inocula for viral agents are reported in Table 2.

Bovine respiratory syncytial virus (BRSV) was removed from liquid nitrogen storage and grown on bovine turbinate (BT) cells for 5 days in 5% CO_2_ at 37°C until cytopathic effect was greater than 50%. Prior to infection of steers tissue culture media except for 10 ml was removed from flasks and transferred to 50 ml conical tubes on ice. Cell sheets then were rapidly frozen at– 80°C to release intracellular virus; media and cells were thawed and decanted into the 50 ml tubes and centrifuged to remove cell debris. Supernatant was stored on ice for transport to the animals. An aliquot was reserved and set up for TCID_50_ viral titer quantitation. The remainder of the supernatant was administered to steers (8.5 ml/animal) using a DeVilbiss (Somerset, PA) nebulizer/air compressor and face mask (Equine Aeromask, Trudell medical International Animal Health).

Bovine herpes virus (IBR) was similarly inoculated onto BT cells and incubated until cytopathic effect (CPE) was obvious at the time that virus was harvested and administered as described for BRSV.

Previously frozen and titered BVDV strain 890 infection stock was grown on BT cells prior to freezing at -80°C. The virus inoculum was defrosted and administered to the steers as described for the other viral pathogens.

Viral control steers received an aerosol of spent but uninfected tissue culture media using a similar nebulizer and facemask system that was designated for use only on the uninfected controls.

### Monitoring of clinical signs

Clinical scores were determined by daily evaluation as in the morning. Either a DVM or veterinary student performed the examinations and provided daily assessment using a scoring system based on a combination of the system described by [4] and one used by Gershwin [5], an adaptation from Collie [6]. The point scoring systems used in the pilot and optimized studies are described in Table 3. Using this system points were accumulated for each abnormal clinical sign and the total number corresponded with severity of disease, such that high numbers were associated with most severe disease. An expanded point system was used in the optimal study (Table 3).

**Table 3:** Scoring system for Clinical Signs.

Study	Parameter Evaluated	Score Scale
Pilot	Rectal temperature	(T -39.5) × 100
	Body Weight	No point value
	Nasal Discharge	*0–3 × 10
	Ocular Discharge	0–3 × 10
	Ear position (droop)	0–3 ×10
	Cough, induced and spontaneous, evaluated separately	0–3 × 10
	Respiratory Rate—breaths/minute	× 1
	Subcutaneous emphysema	Yes or No
	Mandibular lymph nodes	Normal or enlarged
	Lung sounds (left)	0–3 × 100
	Lung sounds (right)	0–3 × 100
	Tracheal sounds	0–3 × 10
Optimal	Rectal temperature (T)	T-39.5° X 40
	Body weight	No point value
	Nasal discharge (each nostril scored separately)	0–3 x 5 (serous), 10 (seromucoid), 15 (mucoid), 20 (mucopurulent)
	Ocular discharge (each eye scored separately)	0–3 x 5 (serous), 10 (seromucoid), 15 (mucoid), 20 (mucopurulent)
	Ear position (droop)	No point value
	Spontaneous cough	0 (no), 40 (yes)
	Induced cough	0 (no), 40 (yes)
	Respiratory Rate (RR)–breaths/minute	RR-40 x 1 =
	Subcutaneous emphysema	0 (no), 100 (yes)
	Mandibular lymph nodes	0 (normal), 50 (enlarged)
	Lung sounds (harsh or quiet; each lung scored separately)	0 (normal), 15 (mild/moderate), 30 (severe)
	Tracheal sounds	No point value
	Crackles (each lung scored separately)	0 (no), 80 (yes)
	Wheezes (each lung scored separately)	0 (n0), 60 (yes)
	Respiratory character	0 (normal), 30 (shallow or deep)
	Respiratory volume (each lung scored separately)	0 (normal), 20 (mild/moderate reduction), 40 (severe reduction)
	Apneustic breathing	0 (no), 30 (yes)
	Mouth breathing	0 (no), 50 (yes)
	Dyspnea	0 (no), 75 (yes)
	Expiratory grunt	0 (no), 60 (yes)
	Biphasic expiration	0 (no), 60 (yes)

*0–3 indicates magnitude, then x indicated weight factor

### Samples

Deep pharyngeal swabs were obtained before infection and on the day of necropsy using a guarded culture instrument with a sheathed polyester swab designed for uterine culture in equine mares (Kalayjian Industries, Inc. Signal Hill, CA). The sheathed swab was inserted into the ventral meatus of the nare and was advanced as far as possible before the internal swab was briefly extended and then withdrawn back into the sheath prior to extraction from the nose. These swabs were delivered to the California Animal Health and Food Safety Laboratory (CAHFS) diagnostic facility, where culture media was inoculated.

Blood was obtained prior to selection of steers for the experiments and viral titers were performed at CAHFS. Sero-negative steers were selected for viral infections. Animals used in the bacterial infection challenges were not screened for antibodies in the pilot study. However, in the optimized study, serum was sent to the laboratory of Dr. A. Confer (Oklahoma State University) for *M*. *haemolytica* and *P*. *multocida* titers which were performed by ELISA [7]. This information was used to select steers with the lowest titers for the optimized study.

### Necropsy

Steers were killed by captive bolt to the head, immediately hung, and exsanguinated by severing the jugular veins. Lungs and other viscera were removed and examined by a board certified veterinary pathologist. In the optimized study, the left lung was lavaged with a solution of Hank’s balanced salt solution. Three 60 ml aliquots were sequentially instilled and withdrawn from the lung using a foal sized stomach tube with internal polyethylene tube, equipped with a 3 way valve. Lavage fluid was stored on wet ice for transport to the laboratory after which the cells were pelleted by centrifugation and frozen at -80°C for future RNA isolation. The supernatant was stored at -20°C.

The respiratory tract was examined: larynx, trachea, bronchi, and lungs were examined and lesions were recorded. Estimation of percent consolidation was made for each lung lobe. The left lung was infused with 10% formalin for histopathological evaluation. From the right lung, tissue samples were taken and immediately frozen in liquid nitrogen for RNA extraction and eventual sequencing. Samples harvested included: tonsil, retropharyngeal lymph node, bronchial lymph node, spleen, liver, and both lesional and normal samples from the uninfused lung lobe. At necropsy, swabs were taken from lung and bronchi and were submitted for bacterial and mycoplasma culture. Immunohistochemistry was performed for BRSV, IBR, and BVDV.

### Immunohistochemistry on Lung Sections

Immunohistochemistry was performed on lung sections for four pathogens: bovine herpes virus -1 (BHV-1), BRSV, BVDV, and *Mycoplasma bovis*. The slides were 3–5 micron sections with an initial unmasking/decloaking step which was either 0.1% Protease type XIV (Sigma P5147) for 15 minutes at 37C for both the BHV-1 and BVDV or the unmasking decloaking step for the BRSV and *Mycoplasma bovis* was 10X Decloaker citrate buffer (Biocare CB910M) for 10 minutes at 121C in pressure cooker. After the unmasking step for BHV-1 the primary antibody mouse anti-BHV 1 Clone F2 (VMRD), diluted 1:3000, was added and incubated for 30 minutes at room temperature (RT). Next the secondary antibody, biotinylated goat anti-mouse (Vector Labs BA9200), diluted 1:200, was added and incubated for 30 minutes at RT. The tertiary reagent (Label) horseradish peroxidase-labeled Streptavidin (Jackson Lab 016-030-084), diluted 1:1000, was added and incubated for 30 minutes at RT. Finally the chromogen AEC, RTU (Dako K3464), was added and incubated for 15 minutes at RT. The slides were counterstained with Mayer’s hematoxylin and blued. Aqueous mounting medium was applied and allowed to harden before permanent coverslips were mounted.

For BVDV staining the protocol was the same as for BHV-1 except the primary antibody was mouse anti-BVDV (Idexx Clone 15.C.5), diluted 1:500.

Staining for BRSV was accomplished after epitope unmasking (see above for both BRSV and *Mycoplasma bovis*). The primary antibody, mouse anti-BRSV Clone 4–2 (Iowa State University), was diluted 1:100 and incubated for 30 minutes at RT. The secondary antibody (same as BHV 1) was biotinylated goat anti-mouse (Vector Labs BA9200), diluted 1:200, and incubated for 30 minutes at RT. The tertiary Reagent (Label): ABC (Vector PK6100) was incubated for 30 minutes at RT and the chromogen and finishing was the same as for BHV 1.

IHC detection of *Mycoplasma bovis* used epitope unmasking as described for BRSV. The primary antibody was *M*. *bovis* rabbit polyclonal (CAHFS Bacteriology #1774 1/31/90) diluted 1:3000 and incubated for 30 minutes at RT. The secondary antibody was anti-rabbit horseradish peroxidase labeled polymer (Dako K4003) incubated 30 minutes at RT. The tertiary reagent (Label) was horseradish peroxidase-labeled streptavidin (Jackson Lab 016-030-084), diluted 1:1000, and incubated for 30 minutes at RT. Last the chromogen, AEC, RTU (Dako K3464), was added and incubated for 15 minutes at RT. The slides were counterstained with Mayer’s hematoxylin and blued. Aqueous mounting medium was applied and allowed to harden before permanent coverslips were mounted.

### Statistical Analysis

In the optimized study, composite scores were obtained on each of four animals for 10 consecutive days (3 prior to challenge and 7 post challenge) for BRSV; 9 consecutive days (3 prior to challenge and 6 post challenge) for IBR; 15 consecutive days post challenge for BVDV; 6 consecutive days (2 prior to challenge and 5 post challenge) for *M*. *haemolytica*; 18 consecutive days (3 prior to challenge and 15 post challenge) for *M*. *bovis*; and 8 consecutive days (2 prior to challenge and 6 post challenge) for *P*. *multocida*. Within each challenge level, four distinct animals were used as controls and composite scores were recorded on challenge and control animals at the same times. Missing values were present in the data leading to a total number of observations (number of days observed × 4 animals—missing values) of 39, 35, 27, 60, 29, and 60 for challenged animals and 37, 35, 28, 70, 32 and 60 for matched controls for BRSV, IBR, BVDV, *M*. *haemolytica*, *M*. *bovis* and *P*. *multocida*, respectively.

For each pathogen we fit the linear model:

$${\text{y}}_{\text{ijkl}}{=\mu +\text{ Treatment}}_{\text{i}}{+\text{ Day}}_{\text{j}}{+\text{Treatment }\times \text{ Day}}_{\text{ij}}{+\text{ Animal}}_{\text{ik}}{+\text{ e}}_{\text{ijkl}}$$

Where y_ijkl_ is a composite score, μ is the overall mean, Treatment_i_ (I = 1, 2) represents the specific pathogen challenge or control, Day_j_ (j = 1, n_j_ and n_j_ are provided above for each treatment) is the day within the consecutive sequence on which the composite score was observed, Treatment × Day_ij_ is the interaction term of interest in this study, Animal_ik_ is the effect of the k^th^ animal (k = 1, 4) within the i^th^ treatment and e_ijkl_ is the residual which is assumed to be normally distributed with zero mean and constant variance. We performed an ANOVA to test the significance of the Treatment × Day_ij_ interaction and, when significant, tested the difference between treatment levels (control vs. pathogen) within each day using a t-test and Bonferroni correction to achieve an overall significance of P < 0.05 for each pathogen.

## Results

### Pilot Study: clinical signs and lung pathology

**Fig 1:**
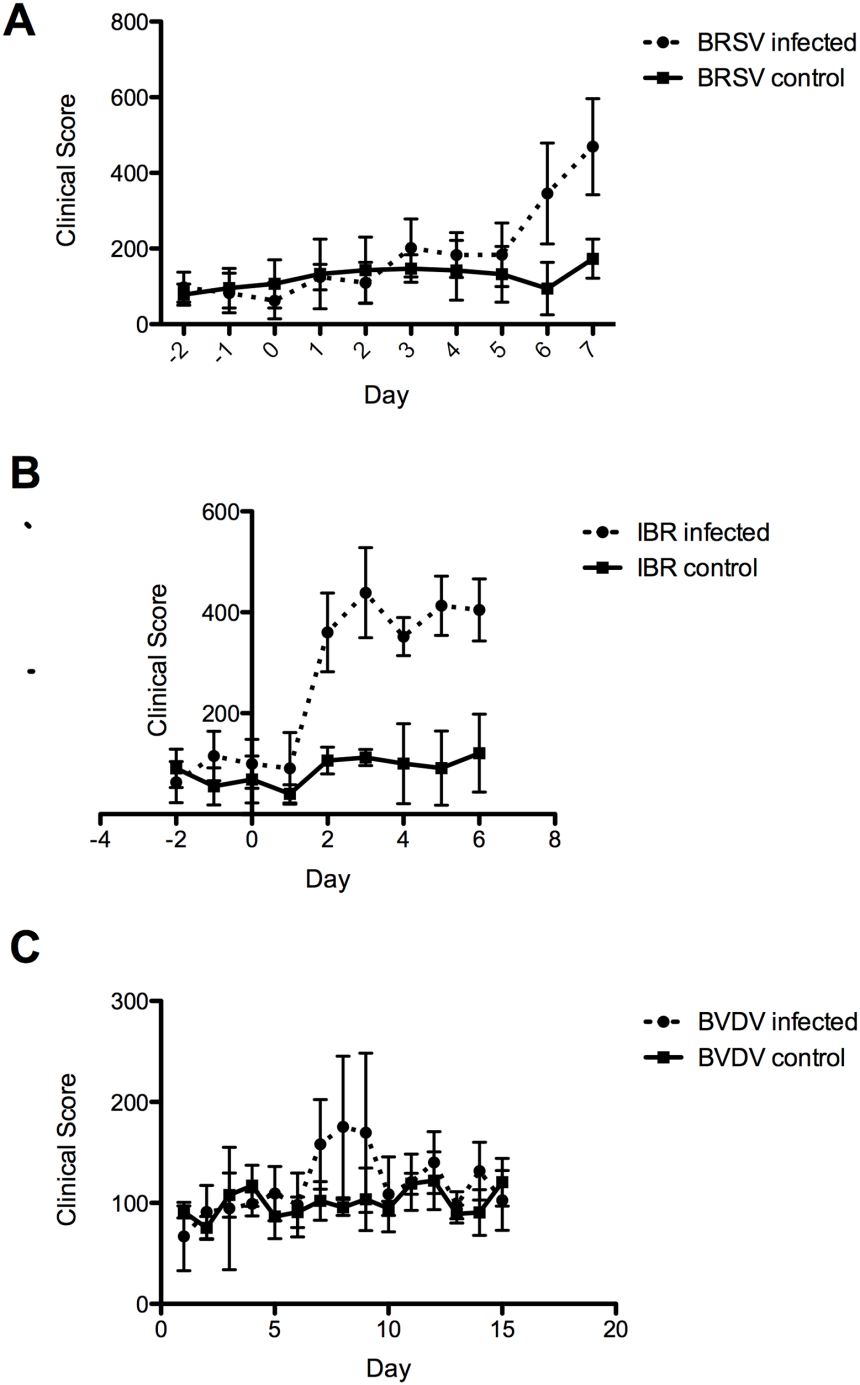
Time course of the mean clinical scores for steers infected with viral pathogens and mock infected controls. Infection with (A) BRSV occurred on day 0 and clinical sign scores are presented for the next seven days. Mean (n = 4) scores were significantly different for both treatment versus day effect and for treatment versus mock infected controls (p<0.005); Bonferroni post hoc test p<0.05. (B) IBR infection occurred on day 0 and mean clinical sign scores are presented for the next six days. Mean (n = 4) scores were highly significantly different from mock infected controls; and treatment versus day effect was significant (Bonferroni test p<0.05). (C) BVDV infection on day 0 was followed until day 15 and mean clinical scores are presented for infected and mock infected controls. The effect of infection (p = 0.0013) and day (p = 0.0071) were significant. Mean clinical sign scores were significantly different for infected and control steers only on day 8 (Bonferroni p<0.058).

BRSV infected steers were necropsied on day 7 when clinical signs were determined to have peaked. These included: fever, increased respiratory rate, cough, and adventitious lung sounds. Lung pathology was consistent with expected lesions. as shown (Fig 1 and S1 Table). Immunohistochemistry on lung sections was positive for BRSV. Day 7 following challenge was determined to be an appropriate time point for necropsy and harvesting of tissues to be used for RNA sequencing.

Infection with IBR virus induced clinical signs consisting of fever, nasal discharge, increased respiratory rate and cough. In the pilot study, clinical scores were greatest on day 6 of infection when necropsy was performed. Immunohistochemistry for IBR virus in the lung was positive for one of the two steers. Day 6 was selected as the appropriate time to harvest tissues for RNA sequencing in the optimized study. Pathology of the respiratory tract included erosions and ulcerations in the trachea and laryngeal mucosa; lung consolidation and expected lung histopathology (Fig 1 and S1 Table).

BVDV infection of the pilot study steers did not produce clinical signs of respiratory disease that differed from pre-infection infection until necropsy on day 8. However, both steers had diarrhea. At necropsy, one steer had 25% consolidation of the right apical lung lobe. Histopathology showed some lymphocytic and neutrophilic accumulations in the lungs of both animals (Fig 1 and S1 Table). Both lungs were negative on immunohistopathology for BVDV. However, gastrointestinal pathology revealed that there was depletion of Peyer’s Patch lymphocytes and there was positive immunohistochemical staining for BVDV in these tissues. These pilot results suggested that a longer duration of infection would be desirable in the optimized study to obtain more consistent clinical and pathological evidence of respiratory disease.

The clinical scores of the pilot study steers infected with *Mannheimia haemolytica* did not differ from those of the mock-infected control animals. Peak mean scores were highest between days 3 and 5 following challenge and were not elevated on day 8 when necropsy was performed. Some lung pathology was observed (S2 Table) but *M*. *haemolytica* was not isolated from the lung of either pilot study animal. These data suggested that the optimized study should include infection with a more virulent *M*. *haemolytica* isolate and necropsy should be performed on day 5 following challenge.

Infection of pilot study steers with *Pasteurella multocida* produced a slight peak in clinical scores between days 2 and 4 and a decline to control levels by day 8 when necropsy was performed. Clinical signs consisted of fever, nasal exudate, and occasional increased lung sounds on auscultation. Only one of the two steers showed lung consolidation. Histopathological observations are described in S2 Table. *Pasteurella multocida* was isolated from the lung of the first steer but not from the steer that lacked lesions (S2 Table). These data suggested that a more virulent isolate coupled with an earlier necropsy day (day 5) would be more effective for induction of disease.

*Mycoplasma bovis* was the final challenge agent utilized in the pilot study. Despite culturing *M*. *bovis* from the lungs and pharynx of both pilot study steers at necropsy on day 7, the clinical signs for these animals did not differ from those of the control steers. No gross lesions were observed in the lungs of one animal and the second animal had some lung consolidation (S2 Table); both animals had minimal histological changes. These results indicated that the duration of infection should be extended and a more virulent *M*. *bovis* isolate should be used to induce a more severe disease prior to necropsy and tissue harvesting.

Lung pathology, gross and histopathology, is tabulated in detail for each calf in S1 Table (viral pathogens) and S2 Table (bacterial pathogens).

### Optimized Study: clinical signs

Infection with BRSV (n = 4) and mock infected controls (n = 4) was performed using the same isolate and schedule as in the pilot study. The infected steers developed clinical signs beginning on day 3, which peaked on day 7 (Fig 1A). Increased temperature, respiratory rate, cough, and adventitious sounds on lung auscultation accounted for most of the score. The clinical signs were significantly different from control (p<0.005, infection), the interaction and day effects were also significant. Bonferroni *post hoc* test: p < 0.05 for days 6 and 7 post-infection.

Steers infected with IBR (n = 4) using the same isolate and infection schedule as in the pilot study, showed a highly significant difference from mock infected controls (n = 4) beginning on day 2 after infection (post-hoc Bonferroni test p < 0.05). Clinical scores stayed elevated through day 6 when necropsy was performed (Fig 1B). These steers showed a significant treatment × day effect.

Infection with BVDV (n = 4) and mock-infected controls (n = 4) was achieved using the same isolate as used in the pilot experiment, but the duration of the experiment was extended to necropsy on day 15. Maximum clinical scores were observed on days 7–9, with little difference from control animals apparent on days 10–15 (Fig 1C). Infection was significant (p = 0.0013) as were day (0.0071), and treatment × day (P<0.027). Treated and control animals differed only on day 8 following infection (Bonferroni *post hoc* test: p< 0.058).

*Pasteurella multocida* infections were performed using a different isolate (Table 2) from that used in the pilot study and necropsy was performed earlier, on day 6. Antibody titers to *P*. *multocida* were evaluated by ELISA and found to be higher than desirable for experimental infection (personal communication A. Confer) on all steers available for the study. This precluded using seronegative steers. Despite the use of a new isolate, the difference in mean clinical score between infected (n = 4) steers and mock infected control steers (n = 4) was not significant on any day post infection.

**Fig 2:**
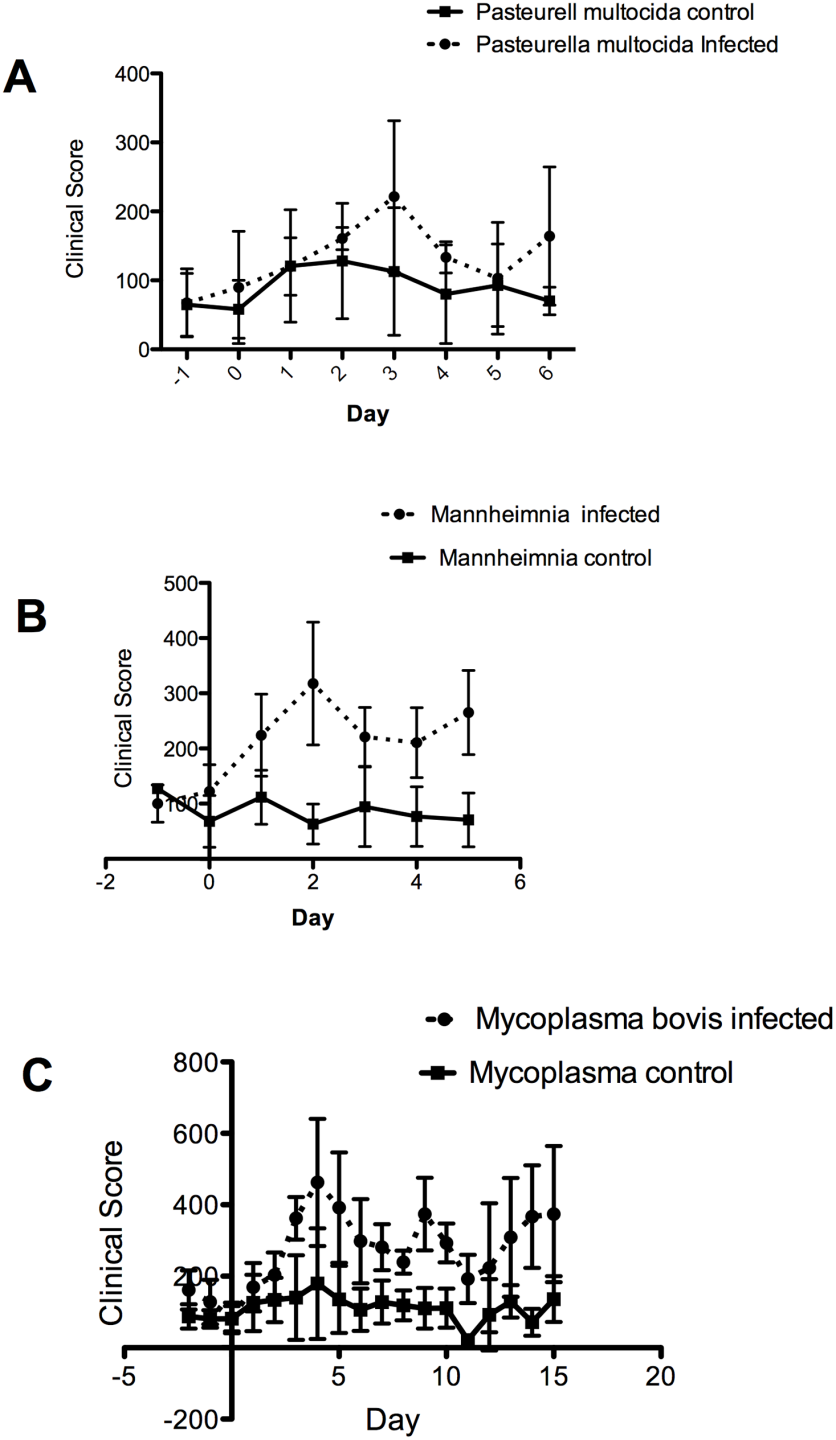
Time course of the mean clinical scores for infected steers infected with bacterial pathogens and mock infected controls. Infection with (A) *Pasteurella multocida* on day 0 was followed until day 6. At no time was the difference in mean clinical scores significantly different between infected (n = 4) and mock infected control steers (n = 4). (B) Day 0 infection with *Mannheimnia haemolytica* was followed for five days. Mean clinical scores were significantly different from day 2 through day five between infected (n = 4) and control steers (p = 0.00135); and the effect of infection and treatment was significant (p = 0.0071), Bonferroni test p<0.05. (C) Infection with *Mycoplasma bovis* on day 0 was followed through day 15 and the difference in mean clinical scores was significant from day 3 through day 15 (Bonferroni p, 0.05). The effect of infection (p = 0.0054) and day (p = 0.0028) was significant.

Infection with *Mannheimnia haemolytica* (n = 4) produced the highest clinical scores on day 2 post-infection and the infected group continued to have elevated scores through necropsy on day 5 (Fig 2B). The difference between infected and mock infected steers (n = 4) was significant (p = 0.00135), infection and day was significant (p = 0.0071) as was the difference between treated and control animals from day 2 onward (Bonferroni *post hoc* test: p< 0.05).

The use of a *Mycoplasma bovis* clinical isolate previously shown to induce experimental diseaseand the extension of the observation period was successful in producing infected animals (n = 4) that had a clinical disease significantly different from mock infected control animals (n = 4) beginning on day 3 and lasting through day 15 (Bonferroni *post hoc* test: p< 0.05) when animals were euthanized for necropsy (Fig 2C). The effects of infection (p = 0.0054), day (p<0.0028) and the interaction of treatment × day were also significant.

### Bacterial isolations

**Fig 3:**
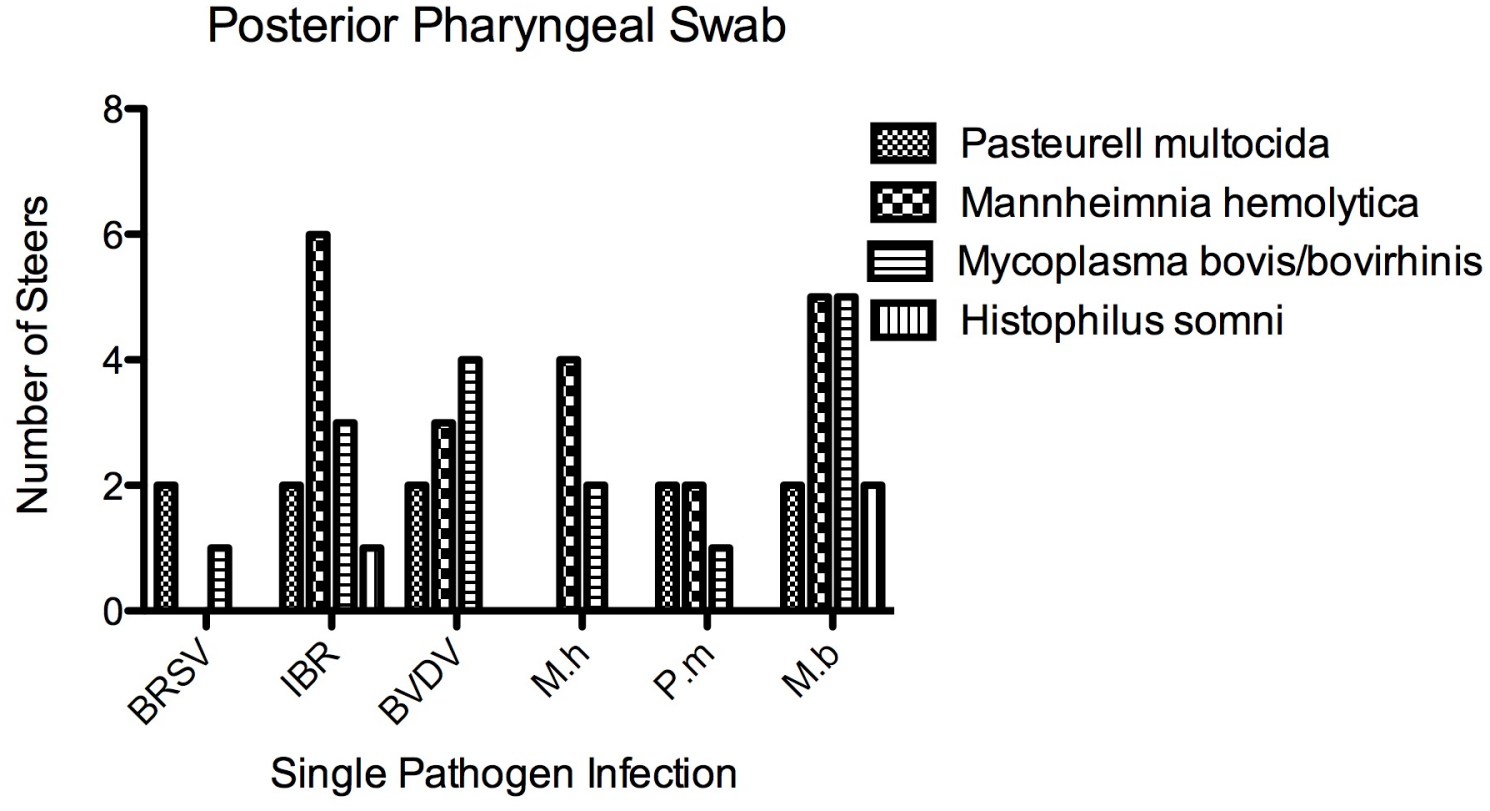
Bacterial isolations from pharyngeal swabs. To evaluate the presence of the BRDC bacterial pathogens in the posterior pharynx of steers before and after infection, sheathed swabs were taken at intervals and at necropsy. The number of steers from each pathogen infection group (x axis) that had each of the bacterial pathogens isolated from the upper respiratory tract either during infection or at necropsy is shown (y axis).

Bacteria and mycoplasma isolated from lungs at necropsy and from deep pharyngeal swabs before and during infection were tabulated in detail for each animal in S1 and S2 Tables, and are summarized in Fig 3. Most BRSV infected steers had mixed flora in the posterior nasal cavity at the start of the experiment; *Pasteurella multocida* was isolated from the nasal cavity of both of the pilot study calves. Yet lungs from five of the six BRSV infected steers were negative for bacteria and mycoplasma at necropsy. *Mannheimnia haemolytica* was present in the nasal cavity in all six of the IBR infected steers by day 6 of infection; several steers had additional organisms present. However, lungs from all six of these steers were negative for bacterial culture at necropsy. The lung swabs cultured for bacteria and mycoplasma in BVDV infected steers were all negative despite the presence of a variety of bacteria and mycoplasma in the posterior nasal cavity detected by swabs either pre-infection of during infection.

*Mannheimia haemolytica* was fairly consistently isolated from the posterior pharynx of the *M*. *haemolytica* infected steers and from the lungs of all four of the optimized study steers. Mycoplasma was consistently isolated from the posterior pharyngeal swabs and lungs of *M*. *bovis* infected steers in the optimized study.

### Pathological Examination

**Fig 4:**
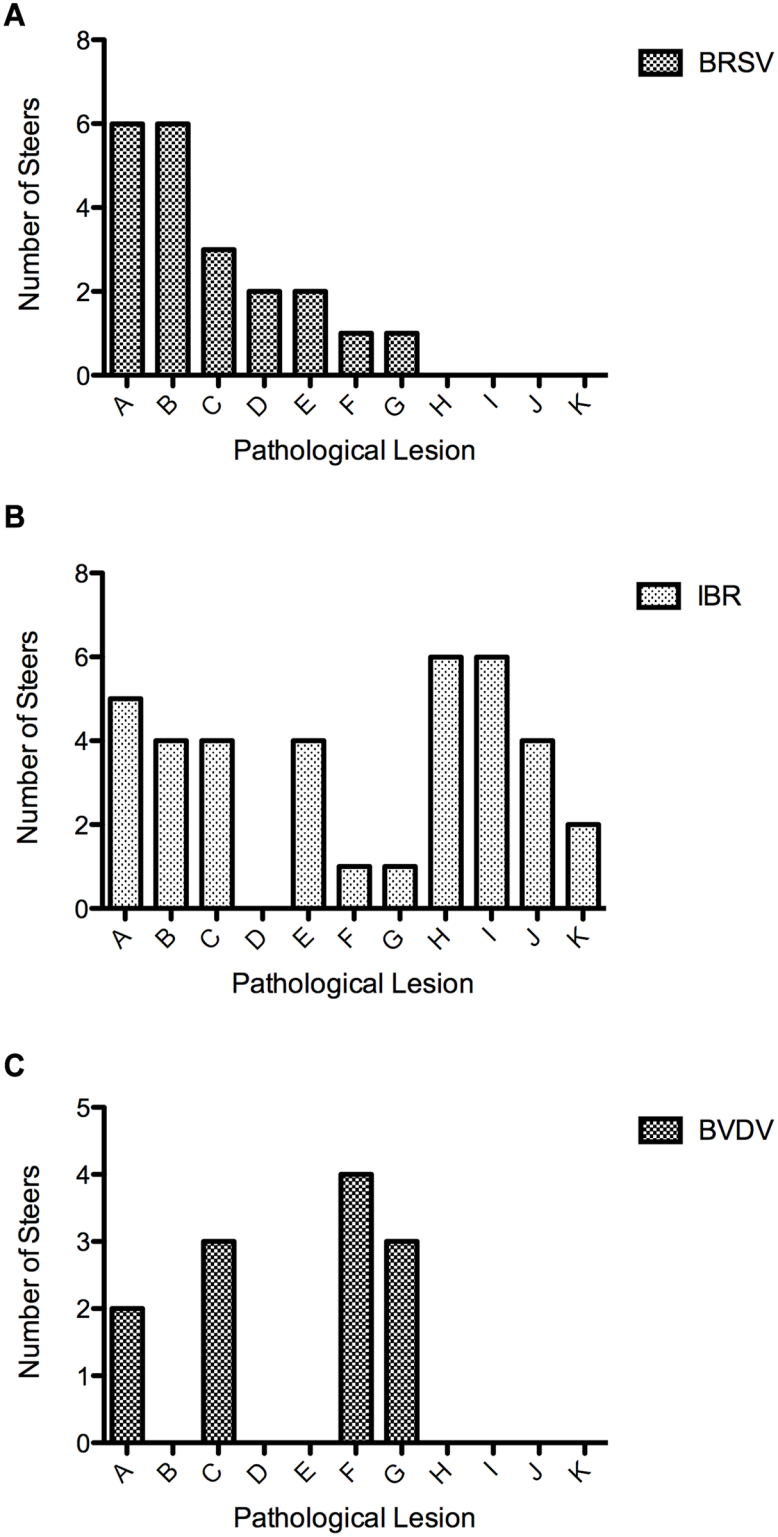
The number of virus infected steers with specific pathological lesions-combined pilot and optimized studies (A-K on x axis) is shown for steers infected with: (A) BRSV, (B) IBR, and (C) BVDV. Lesions are: A) total lung consolidation >1%, B) neutrophilic proliferative necrotizing bronchiolitis/bronchitis, C) supperative bronchopneumonia, D) syncytial formation, E) neutrophilic alveolitis, F) plasmacytic bronchitis/bronchiolitis, G) bacterial culture positive, H) ulcerative rhinitis, I) laryngeal ulceration or erosion, J) tracheal ulceration, K) intranuclear inclusion bodies.

**Fig 5:**
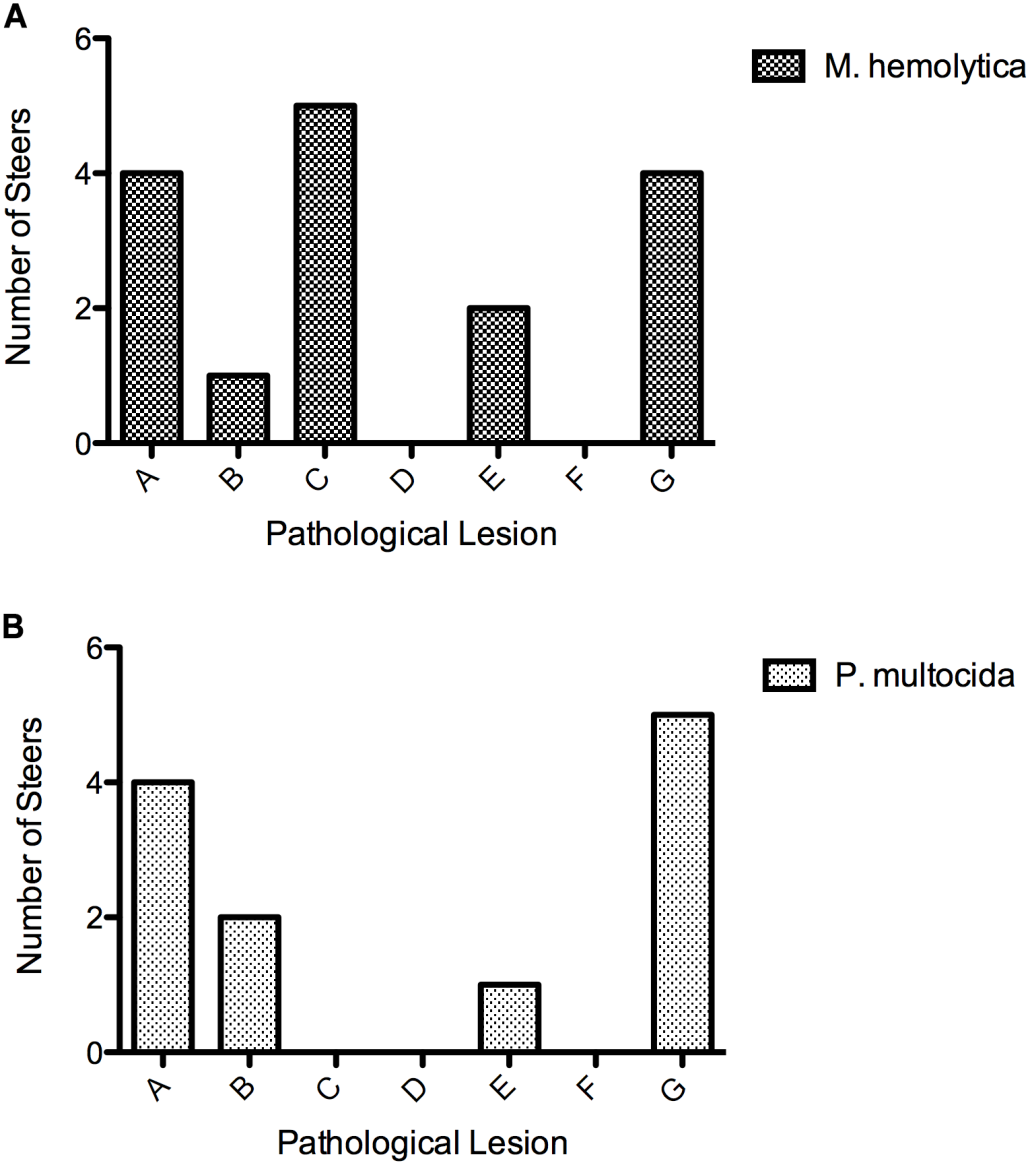
The number of bacterial infected steers with specific pathological lesions (A-F)-combined pilot and optimized studies. + on x axis) is shown for steers infected with: (A) *Mannheimnia haemolytica*, (B) *Pasteurella multocida*, and (C) *Mycoplasma bovis*. Lesions are: A) total lung consolidation <1%, B) neutrophilic bronchitis/bronchiolitis, C) subacute necrotizing fibrinocellular lobar pneumonia and pleuritis, D) neutrophilic bronchopneumonia with necrosupperative obliterating bronchiolitis/bronchitis, E) lymphocytic bronchitis/bronchiolitis, F) lymphoid hyperplasia.

**Fig 6:**
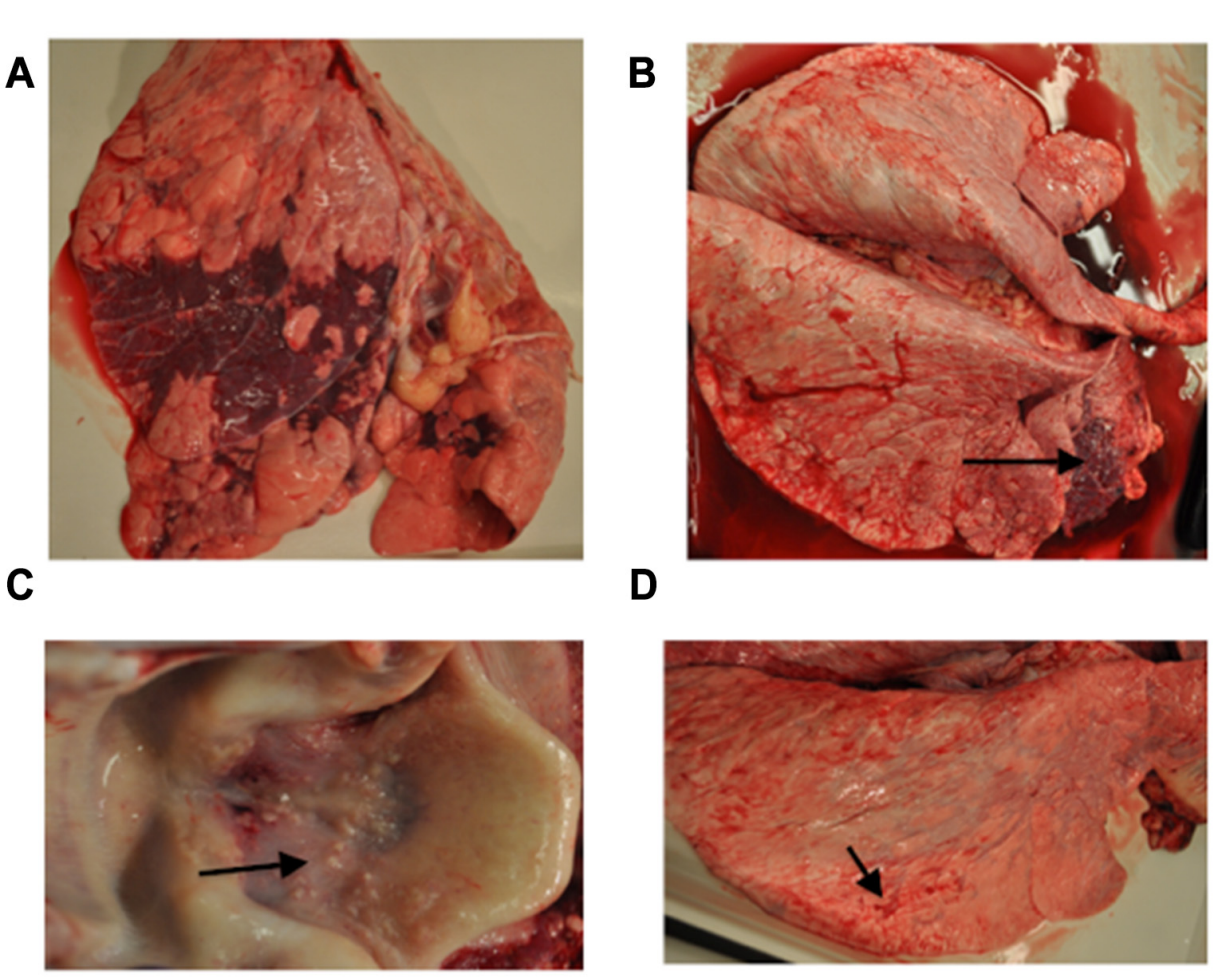
Examples of gross pulmonary pathology from virus infected steers: (A) BRSV infected lung showing lobar lung consolidation (dark areas), (B) IBR infected lung with consolidation (arrow) and scattered areas of atelectasis and necrosis, (C) IBR infected larynx showing areas of ulceration and erosion (arrow), and (D) BVDV lung showing minimal pathology with scattered small areas of consolidation (arrow).

**Fig 7:**
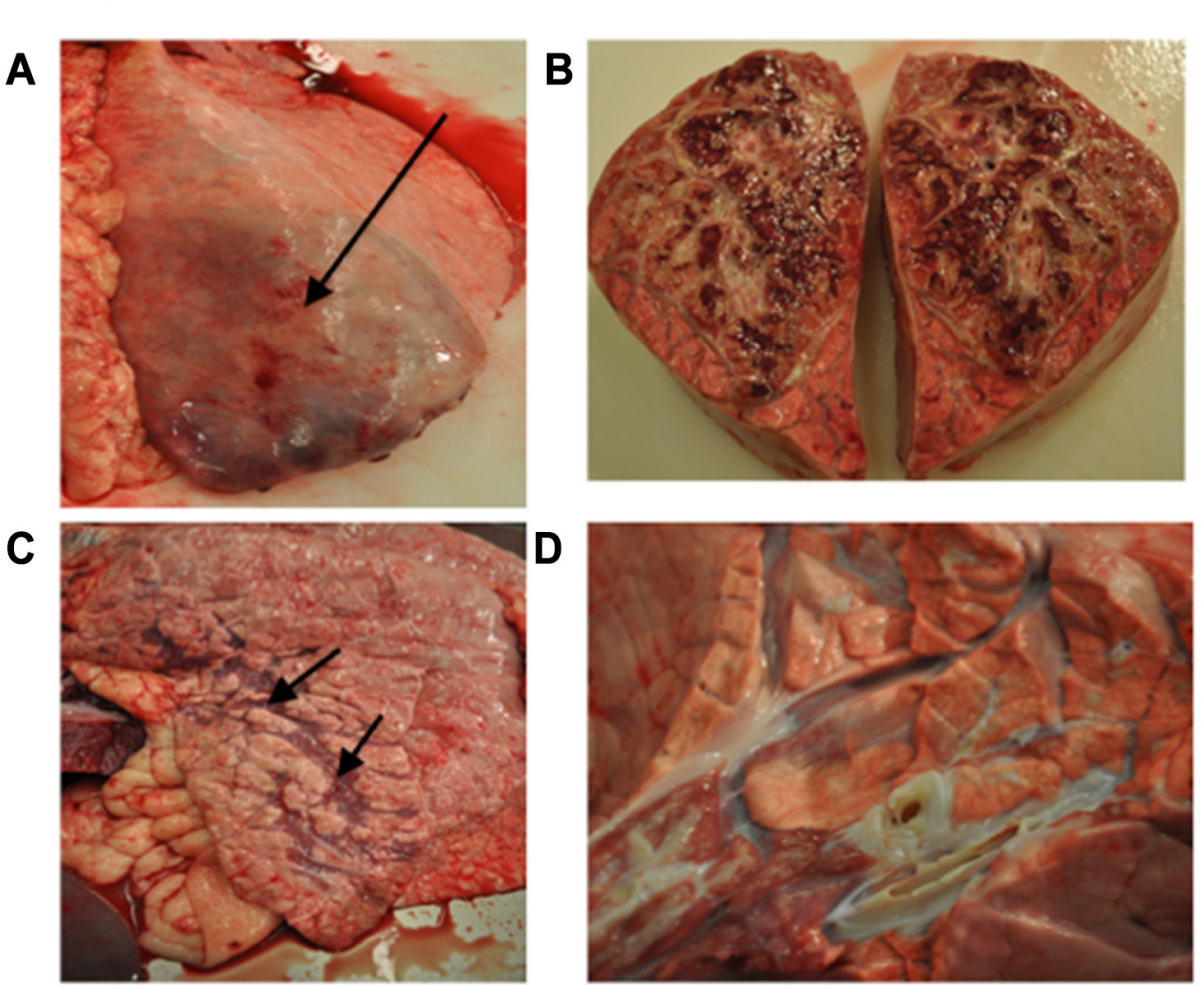
Examples of gross pathology from bacterial infected steers: (A) *Mannheimnia haemolytica* lung showing consolidation (arrow) and (B) Lung from *Mannheimia haemolytica* infected steer demonstrating irregular dark red consolidated regions bordered by light grey inflammatory infiltrate with expanded interlobular septa. (C) *Pasteurella multocida* infected lung with minimal lesions consisting of scattered areas of consolidation (arrows), and (D) Lung from *Mycoplasma bovis* infected steer demonstrating a focal region (lower left) of consolidation associated with bronchitis with luminal exudate.

Major histological lesions of lungs are reported in Fig 4 for each infection group and for individual animals in S1 Table (viral pathogens) and in Fig 5 and S2 Table (bacterial pathogens). Representative gross lung lesions are presented in Fig 6 for viral pathogens and in Fig 7 for bacterial pathogens. For those pathogens that induced characteristic pathological lesions in the pilot study steers, tissue samples were retained for RNA extraction, thereby increasing the animal sample number for these pathogens to six.

**Fig 8:**
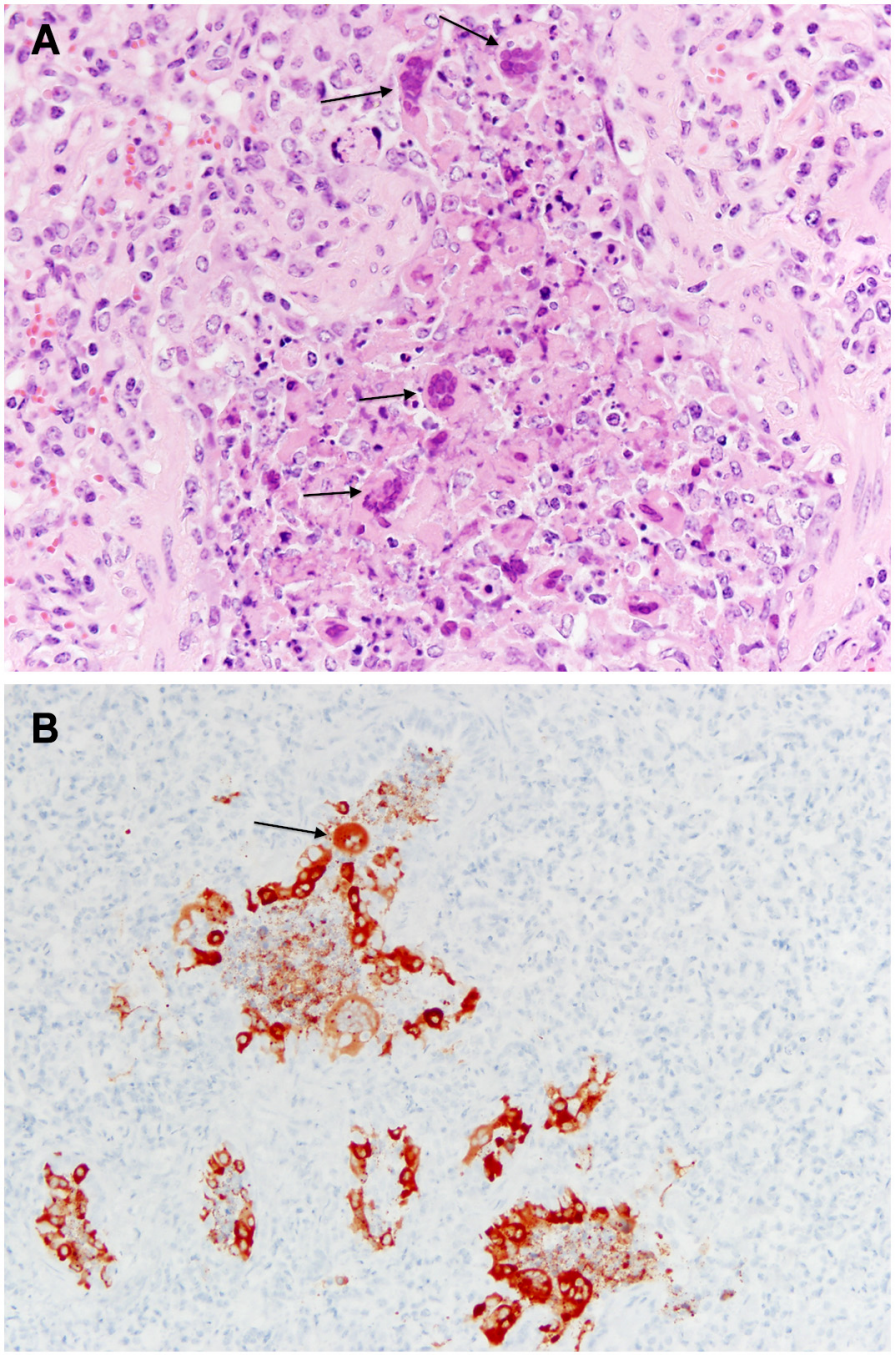
Histopathology of lung from BRSV infected steer. (A) Bronchiolitis with epithelial necrosis and numerous syncytia (arrows), (B) Immunohistochemistry stain showing positive stain for BRSV in bronchioles (brown color and arrow).

In the six BRSV infected steers, the lung consolidation varied from an overall 10% to 40%, with differences in individual lobe consolidation within and between steers. As shown in Figs 4A and 6A all BRSV infected steers had consolidation. Atelectasis was present in some lung lobes and generalized emphysema was apparent in one steer. The frequency of the most characteristic histological lesions is also documented in Fig 4A. Histological examination showed neutrophilic or pleocellular proliferative necrotizing bronchitis and bronchiolitis in all (6 out of 6) infected steers. Immunohistochemistry for BRSV was positive in all 6 lungs. There were syncytial cells and neutrophilic alveolitis noted in 2 of the 6 steers bacteria were culture from the lungs of only one of the six steers. Examples of characteristic BRSV histological lesions are shown in Fig 8A. Immunohistochemical staining for BRSV is shown in Fig 8B.

**Fig 9:**
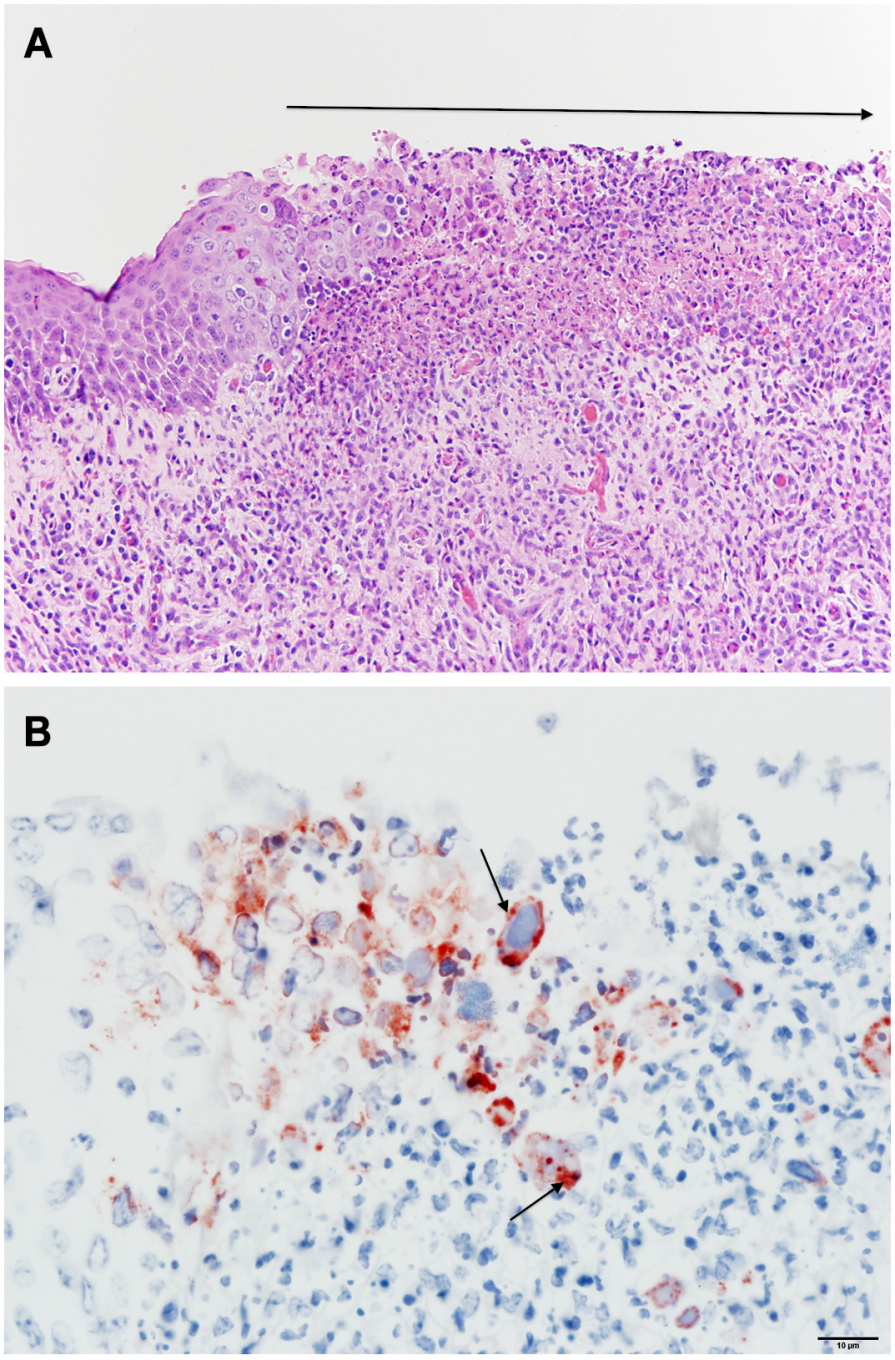
Histopathology of pharynx from IBR infected steer. (A) Focal ulcerative pharyngitis with epithelial necrosis (area indicated by length of the arrow), (B) Immunohistochemistry of lesions demonstrating positive cytoplasmic staining for IBR, indicated by arrows and red/brown color.

Lungs from six infectious bovine rhinotracheitis virus infected steers showed 5% to 15% overall lung consolidation (Fig 6B); with disseminated atelectasis and necrosis present in lungs from two of the six steers Ulcerative rhinitis, laryngitis (6 out of 6), and tracheitis (4 out of 6) were present in (Fig 4B) and shown in Fig 6C. On microscopic examination, there were ulcers and erosion with neutrophilic infiltrates in the larynx and trachea; nuclear inclusions were demonstrated in two of six steers Lung histology showed lymphoplasmocytic bronchitis, bronchiolitis, alveolitis, and some disseminated bronchopneumonia (Figs 4B and 9A). Five of the six IBR infected lungs were stained positive for IBR virus with immunohistochemistry (Fig 9B).

**Fig 10:**
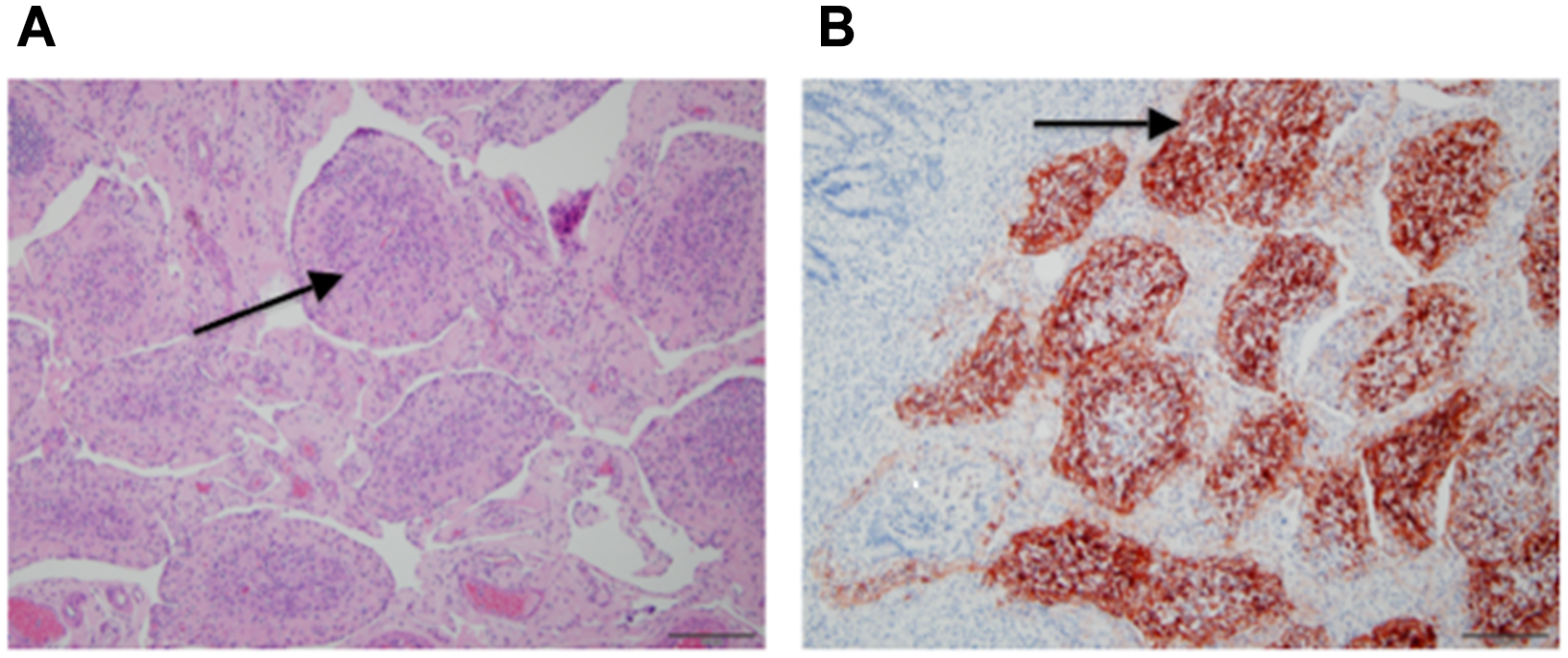
Histopathology of the ileum from a BVDV infected steer. (A) shows section of ileum with depletion of lymphocytes from Peyer’s patches (arrows), and (B) Immunohistochemistry showing positive staining for BVDV in ileum of infected steer (arrow showing brown color).

Consolidation varied from 0% overall to 25% in the six BVDV infected steers. Overall gross pathology was minimal. Histological examination showed diffuse lymphocytic and neutrophilic infiltrates in tracheal mucosa and surrounding bronchi in the lung; atelectasis was common and focal bronchopneumonia was present in some. Individual differences in lung histology accompanied the presence of *Mycoplasma bovis* in the lungs of three steers detailed in S1 Table. In the ileum, Peyer’s patches showed characteristic lymphoid depletion (Fig 10A) and positive staining by immunohistochemistry for BVDV (Fig 10B).

**Fig 11:**
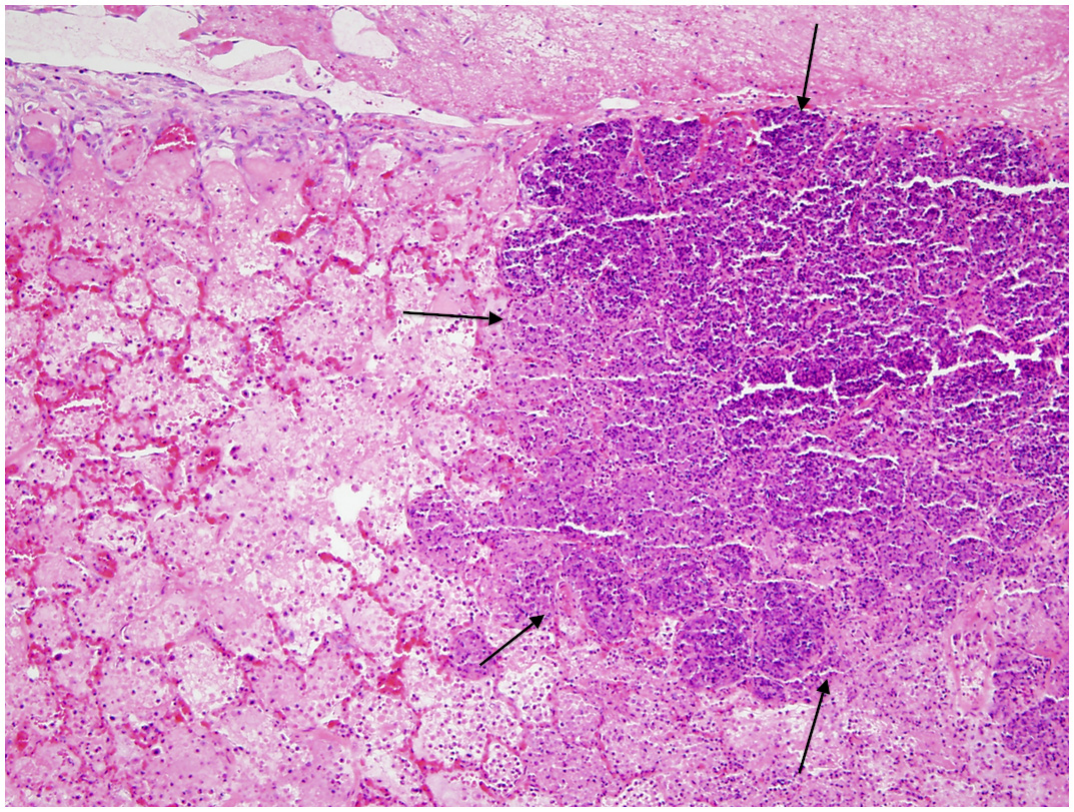
Histopathology of the lung from a *Mannheimnnia haemoytica* infected steer. Lobar pneumonia with irregular multilobular zones of coagulation necrosis and fibrinocelluar exudation bordered by dense bands of necrotic leukocytes (arrows).

**Fig 12:**
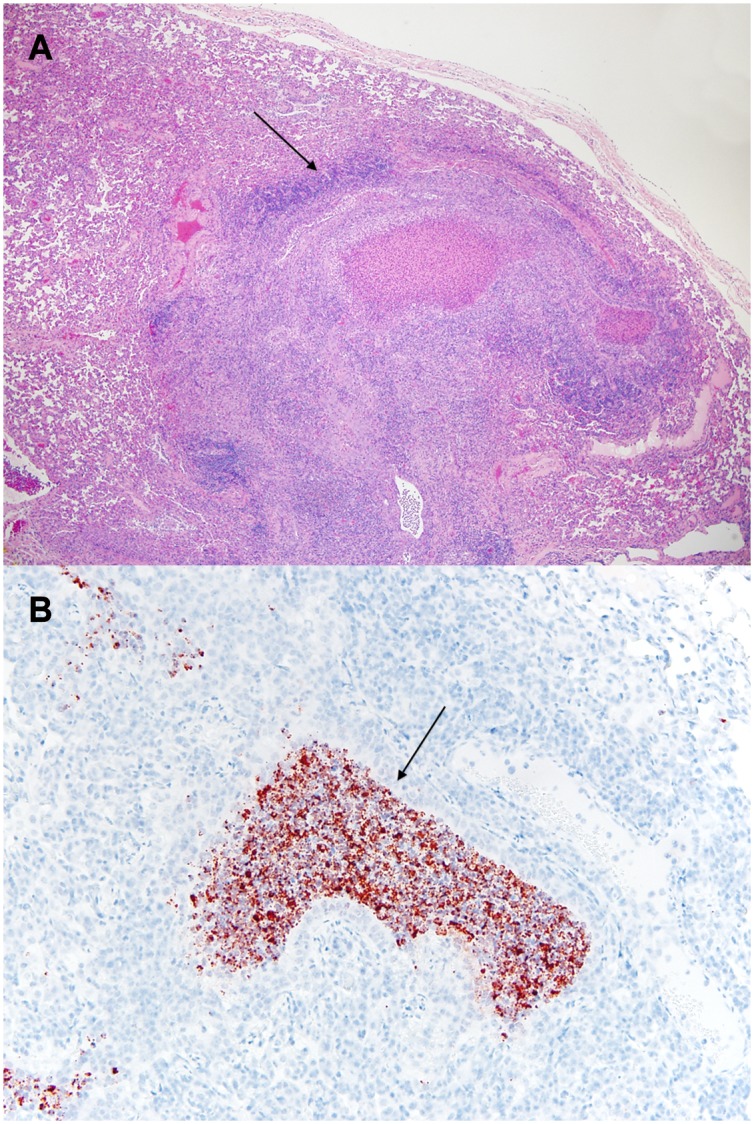
Histopathology of the lung from *Mycoplasma bovis* infected steer. (A) necrosuppuratve bronchiolitis with peribronchiolar mixed leukocytic infiltrate (arrow), (B) Immunohistochemistry of lesion demonstrating positive staining for *Mycoplasma bovis* in the bronchiolar luminal exudate (brown color and arrow).

Gross and histological findings from bacterial pathogens are described in Figs 5, 11 and 12. Lung consolidation varied from 0 to 30%, with some lungs showing primarily atelectasis (Fig 8A and 8B). *Mannheimia haemolytica* was isolated from all four of the *Mannheimnia haemolytica* infected steers in the optimized study and from none of those in the pilot study. The lung histology from those four steers showed subacute necrotizing fibrinocellular lobar pneumonia with coagulation necrosis and pleuritis and is described for each animal in Fig 11 and S2 Table.

*Pasteurella multocida* produced minimal gross lung pathology (Fig 7C). However, histopathology of the pilot study steers showed multiple lesions, including chronic bronchopneumonia and alveolitis (Fig 5). All four of the optimized study steers had *Mycoplasma bovis* isolated from their lungs at necropsy, making this an inadvertent mixed pathogen infection. Histological lesions of bronchiolitis (lymphoid-lymphofollicular) could be attributed to the *M*. *bovis*.

Lungs from two *Mycoplasma bovis* infected steers from the pilot study were examined and found to have no gross lesions. Three steers from the optimized study were examined. The fourth steer was euthanized early due to extreme disease and was eliminated from the study (*M*. *haemolytic*a was diagnosed as the causal agent at necropsy). Consolidation varied from 3% to 35% in the remaining steers. *M*. *bovis* was cultured from all three (Figs 5C and 12A and S2 Table). Necrosuppurative obliterating bronchiolitis and lymphoid hyperplasia were consistent findings. Immunohistochemistry for Mycoplasma bovis was positive and is shown in Fig 12B.

Examination of lungs from bacterial and viral control calves showed no gross lesions and there was no bacterial growth from the lungs at necropsy. Mild bronchus associated lymphoid tissue (BALT) hyperplasia was present on microscopic examination of lungs from the viral control steers (58, 101) and one bacterial control steer (102).

## Discussion

Bovine respiratory disease complex is not a single disease; it is a syndrome caused by the interaction of several viral and bacterial infections that together cause clinical disease and lung pathology. Each pathogen is unique in its interaction with the immune system of the bovine host and the particular immune responses that are most protective for each are not necessarily identical. For many years, the approach to prevention of BRDC has been the development of vaccines for the various component pathogens and altering modes of cattle processing and shipping to decrease stress. The use of prophylactic antibiotic therapy is also common in many conditioning programs. Current regulatory efforts to decrease the usage of antibiotics in livestock emphasize the need to find alternate means of controlling BRDC. The tools for analysis of gene expression and the identification of heritable resistance traits are now available and make it possible to evaluate gene expression in response to environmental stimuli, such as infection with a particular pathogen. The work described herein was performed to provide tissues from single pathogen challenge for each of the most important BRDC pathogens for the purpose of RNA sequencing. Identification of genes involved in the response to each pathogen provides the framework for the recognition of gene variants in larger populations that can serve as potential candidates for selective breeding of cattle more resistant to the agents of BRDC. Ongoing work by our group focuses on evaluation of the genes differentially expressed in each of these single pathogen infections. Initial studies on immunological responses using RNA sequence analysis of bronchial lymph nodes of these animals is presented [3].

Each single pathogen infection had a unique time course for the disease process in each of the six pathogens. Infections with the viral pathogens BRSV, IBR and BVDV each showed a different time course. Necropsy of steers on day 7 of BRSV infection showed typical lesions and the presence of virus in the lung. In contrast BVDV took 15 days to show maximum clinical disease and the presence of virus in the ileum. Experimental infection with *M*. *haemolytica* caused clinical signs to appear on day 2. In the BRDC, the viral infection usually precedes the bacterial infection of the lung making it difficult to identify the initiation of the bacterial infection. These observations underscore the need for single infection data to assure optimum tissue sampling for gene expression studies and for understanding the pathogenesis of each unique host/pathogen interaction.

The interaction of pre-existing pharyngeal flora with experimental infection showed that existence of low numbers of other bacterial organisms, in the upper respiratory tract, is not likely to alter gene expression data obtained from lung or lung associated lymph nodes of experimentally infected steers. Culture of the posterior pharyngeal flora was performed with sheathed swabs. The data is presented in Fig 3 and S3 Table. Culture of the posterior pharynx of the six BRSV infected steers prior to infection with BRSV showed that only one animal was negative for bacteria and mycoplasma. Despite the presence of bacteria in the pharynx at the time of infection in five steers, bacteria were isolated from the lung of only one steer at necropsy. This isolate was described as rare (few organisms found) and it was the same genus and species (*P*. *multocida)* that was isolated from the pharynx prior to infection. The same trend was apparent for IBR: *M*. *haemolytica* was present in the pharynx on either day 0 or day 6 in all six steers, yet the lung was negative at necropsy for these bacteria in all IBR infected steers. BVDV infected steers show a different pattern: four out of six steers had mycoplasma present in the pharyngeal region prior to infection and three of those four had *M*. *bovis* cultured from the lung at necropsy. Bacteria were present on day 0 in the pharynx of four of the six steers (these included *P*. *multocida* and *M*. *haemolytica*); yet none of these were cultured from the lung at necropsy.

Necropsy observations for all three viral pathogens, *M*. *haemolytica*, and *M*. *bovis* included classic lesions, as previously described for these agents [8,9,10,11,12,13]. BRSV lesions were classic, as we have shown previously with this isolate. These included multilobular consolidation with histiological lesions of bronchiolitis with epithelial necrosis and syncytia [14]. For infectious bovine rhinotracheitis we saw the expected nasal, laryngeal, and tracheal mucosal erosion and ulceration. Lung involvement in steers that were negative for bacteria showed lymphocytic/plasmocytic pneumonia and pleocellular bronchitis accompanied by the identification of inclusion bodies which was clearly representative of field cases of IBR. Finally the classic BVDV lesions in Peyer’s Patches, the presence of lymphoid depletion, indicates that the BVDV infection targeted the expected cells and confirmed that the infection was productive and representative of field cases.

The main lesions noted in *M*. *haemolytica* infected steers were fibrinosuppurative or fibrinocellular pneumonia with or without pleuritis. Histological lesions included coagulation necrosis and fibrinocellular exudation. Bronchiolitis obliterans or chronic obliterating bronchitis were commnly associated with *P*. *multocida* infection in spite of the lack of significant clinical signs. In those cases from which *M*. *bovis* was isolated from the lung lymphofollicular bronchitis and bronchiolitis were common findings. Peribronchial lymphoid hyperplasia with a mixed leukocyte infiltrate was the singular most predominant lesion associated with *M*. *bovis* infection. As shown in S2 Table, each steer had a variety of pathological abnormalities and the presence of neutrophil accumulations in interstitial tissues and in airways was a common finding. It is neither unexpected nor novel to have observed this pathology; in fact it is assuring to know that the experimental infections mimicked to a large degree natural disease findings.

As recognized in the field BRDC is a multi- pathogen disease with host- pathogen and pathogen-pathogen interactions intensified by environmental factors that create stress. Thus, for the bacterial infections in the absence of viral infection and with none of the usual stress factors, the nature of the disease was not as severe as generally occurs when the pathogen is combined with the other inciting factors in BRDC. To accomplish the goal of understanding which genes are activated in response to each pathogen alone, it was necessary to perform single pathogen challenge and this goal was achieved for six of the seven major BRDC pathogens.

## Summary and Conclusions

Experimental infection of bovine steers was successfully performed with each of three viral, two bacterial, and one mycoplasmal agents of BRDC after optimizing conditions for infection. For five of the six pathogens clinical sign scores were significantly different from those of mock-infected control steers. Lung pathology was consistent with expected lesions for each of those pathogens. Herein we have presented optimized protocols for induction of disease by each of the major BRDC pathogens in the absence of confounding conditions or infections. In the *P*. *multocida* steers, who failed to show a significant difference from control steers, the presence of high antibody titers prior to infection was a potential protective factor and serves to illustrate the benefit of humoral immunity against this pathogen.

## Supporting Information

S1 Table Summary of Lung Pathology for Viral Pathogens. (DOCX)

S2 Table Summary of Lung Pathology for Bacterial Pathogens. (DOCX)

S3 Table Bacterial Isolations from Lung and Posterior Pharyngeal Swabs (DOCX)
