# Supplementary material for: Single Pathogen Challenge with Agents of the Bovine Respiratory Disease Complex
Source: PLoS One. 2015 Nov 16;10(11):e0142479. doi: 10.1371/journal.pone.0142479 (PMC4646450; doi:10.1371/journal.pone.0142479)
Supplement: S1 Table — (DOCX) [file pone.0142479.s001.docx]

**Table S1.** Summary of Pathology for Viral Pathogens

**A. Bovine Respiratory Syncytial Virus (BRSV)**

|  | **Gross pathology (all % values are estimates)** | **Histopathology/culture** |
| --- | --- | --- |
| 18 | Bilateral widely distributed focal to coalescing consolidation varying from 10 to 20 % in affected lobes. Overall there is less than 10% consolidation; entire lung shows expansion of parenchyma suggestive of emphysema. | Moderate bilateral multilobular consolidation with neutrophilic proliferation necrotizing bronchitis/bronchiolitis with syncytial formation and neutrophilic alveolitis  Bacterial culture negative. BRSV IHC* positive. |
| 33 | Extensive widespread consolidation bilaterally, varying from 10 to 30% in affected lobes. Overall the lung is 15 – 20% consolidated. Throughout the pink aerated lung there is expansion with gas consistent with generalized emphysema | Bilateral multilobular consolidation with neutrophilic proliferative necrotizing bronchitis/bronchiolitis with syncytial formation and neutrophilic alveolitis. Bacterial culture – rare *P. multocida*. BRSV IHC positive. |
| 74 | Bilateral mild multifocal areas of atelectasis and consolidation; overall less than 5% of the lung is affected with consolidation. | Disseminated necrotizing pleocellular proliferative bronchitis/bronchiolitis with lobular atelectasis and minimal suppurative bronchopneumonia. Bacterial culture negative. BRSV IHC positive. |
| 77 | Bilateral areas of lobular consolidation with mucoid exudate in airways. Right lung consolidation varied from 80% apical, 30% caudal, 80% accessory; and 10 to 50% of left lung lobes. Overall 40% of the lung parenchyma is involved. | Disseminated necrotizing pleocellular proliferative bronchiolitis with regional suppurative bronchopneumonia. Bacterial culture negative. BRSV IHC positive. |
| 92 | Bilateral mild multifocal lobular areas of atelectasis and consolidation, mucoid airway exudate. Overall less than 5% of the lung is involved. | Disseminated necrotizing neutrophilic proliferative bronchitis/bronchiolitis with lobular atelectasis and minimal suppurative bronchopneumonia. Bacterial culture negative. BRSV IHC positive. |
| 116 | Multilobular consolidation: 40% of the right apical; 25% right middle, <10% of right caudal, 25% accessory lobe, 5% left apical, 25% left middle, 15% left apical. | Disseminated necrotizing pleocellular proliferative bronchitis/bronchiolitis with mild neutrophilic bronchopneumonia; minimal chronic lymphoplasmacytic to lymphofollicular bronchiolitis. Bacterial culture negative. BRSV IHC positive. |

* IHC = Immunohistochemistry

**B. Bovine herpes virus -2 (Infectious Bovine Rhinotracheitis or IBR)**

| **Animal** | **Gross Pathology** | **Histopathology/culture** |
| --- | --- | --- |
| 81 | Lung: right caudal lobe 10% consolidated, left middle lobe is 25% consolidated and left caudal lobe 20%. Overall 5% of the lung is consolidated. | Nasal mucosa, Laryngeal and tracheal mucosa are essentially as described for #138.  Lung: Interstitial infiltrate around airways in lung. Bacterial culture negative. IHC negative |
| 138 | Lung: mild bilateral ventral consolidation involving right caudal (10%) and multilobular consolidation involving 75% of accessory lung lobe; left caudal lobe 25% consolidation. Overall 15% of the lung is consolidated. | Nasal mucosa: mild intraepithelial lymphocyte infiltrate and lymphoplasmacytic lamina propria infiltration. Laryngeal mucosa: focal erosion and ulceration, leukocytic infiltrates. Trachea: loss of ciliated epithelium with focal mucosal ulceration. Lung: widespread lymphoplasmocytic bronchitis/bronchiolitis with neutrophilic luminal exudate. Bacterial culture negative. IHC positive |
| 59 | Rhinitis, laryngitis, tracheitis with regional unilateral pulmonary consolidation and disseminated bilateral pulmonary hemorrhage/atelectasis/necrosis involving 5% of the lung. | Necrosuppurative ulcerative rhinitis and laryngitis and disseminated pleocellular bronchopneumonia with fibrinohemorrhagic bronchiolitis and alveolitis with focal suppurative bronchopneumonia. Bacterial culture: Histophilus somni and Mannheimia haemolytica isolated. IHC positive |
| 70 | disseminated multifocal pulmonary hemorrhage/atelectasis/necrosis involving 30% of the lung | Necrosuppurative ulcerative rhinitis and laryngitis with intranuclear inclusions and disseminated bronchopneumonia with pleocellular fibrous bronchiolitis and alveolitis  Bacterial culture: negative, IHC positive |
| 76 | Rhinitis, laryngitis and tracheitis and mild regional unilateral consolidation with multifocal disseminated lobular atelectasis/consolidation involving less than 10% of the lung. | Necrosuppurative ulcerative rhinitis, laryngitis and tracheitis with intranuclear inclusions and focal lobar pleocellular bronchopneumonia with disseminated necrosuppurative bronchiolitis and alveolitis. IHC positive |
| 100 | Laryngitis, tracheitis and mild bilateral multilobular atelectasis/consolidation involving 5% of the lung. | Multifocal necrosuppurative ulcerative rhinitis, laryngitis, and tracheitis and disseminated bronchopneumonia with fibrinohemorrhagic, pleocellular necrotizing bronchiolitis and alveolitis. IHC positive |

**C. Bovine Virus Diarrhea Virus (BVDV)**

| **Animal** | **Gross pathology** | **Histopathology/culture** |
| --- | --- | --- |
| 82 | Minimal brown multilobular discoloration in cranial right lobe and left dorsal caudal lobe; no consolidation | Trachea: patchy lymphohistiocytic accumulations in submucosa and light diffuse neutrophilic exocytosis.  Lung: Dense follicle of lymphocytes within a lobule peripherally, patchy atelectasis. Bacterial culture negative. BVD IHC negative in lung. |
| 128 | 25% red-brown consolidation of right apical lobe. Mucoid grey exudate in airways | Trachea: diffuse lymphocytic and neutrophilic infiltrates. Lung: and exocytosis.  Lung: dense lymphocytes, plasma cells and neutrophils around bronchi. Exocytosis of neutrophils through mucosal epithelium Bacterial culture negative. BVD IHC negative in lung |
| 86 | Focal pleural adhesion, right apical lobe; regional multifocal atelectasis | Bronchitis, bronchiolitis: lymphoplasmocytic and lymphofollicular, minimal focal bronchopneumonia and atelectasis.  Peyer’s patch depletion  Bacterial culture negative  BVD IHC positive in ileum |
| 98 | Unilateral apical consolidation of right lung (1%). | Bronchitis, bronchiolitis, lymphoplasmacytic, lymphofollicular, with caseous pyogranulomatous pneumonia  *Mycoplasma bovis* isolated from lung  Peyer’s patch depletion  BVD IHC positive in ileum |
| 138 | Minimal regional lobular consolidation, right apical and left middle lobes (1%) | *Bronchitis, bronchiolitis, lymphoplasmacytic, lymphofollicular with focal bronchopneumonia and obliterating bronchiolitis*  *Mycoplasma bovis* isolated from lung  Peyer’s patch depletion  BVD IHC positive in ileum |
| 139 | Mild unilateral right apical consolidation of right apical and accessory lung lobes affecting 2% of the lung | Bronchitis, bronchiolitis lymphoplasmacytic and lymphofollicular, regional with pleocellular focal bronchopneumonia with obliterating bronchiolitis and focal necrotizing pyogranulomatous pneumonia  *Mycoplasma bovis* isolated from lung  Peyer’s patch depletion  BVD IHC positive in ileum |
