# Supplementary material for: Single Pathogen Challenge with Agents of the Bovine Respiratory Disease Complex
Source: PLoS One. 2015 Nov 16;10(11):e0142479. doi: 10.1371/journal.pone.0142479 (PMC4646450; doi:10.1371/journal.pone.0142479)
Supplement: S2 Table — (DOCX) [file pone.0142479.s002.docx]

**Table S2.** Summary of Lung Pathology for Bacterial Pathogens

**A. *Mannheimia haemolytica***

| **Animal** | **Gross pathology (all % values are estimates)** | **Histopathology/culture** |
| --- | --- | --- |
| 73 | Mild bilateral multilobular atelectasis in left middle (<10%), right apical (<10%) and right middle (<10%) | Lymphonodular  bronchiolitis/bronchitis with multifocal, neutrophilic bronchitis and alveolitis and bronchiolitis obliterans, no bacteria isolated from lung |
| 75 | Atelectasis in the left middle lung (20%); 5% lobular atelectasis in the right middle lung and  focal lobular atelectasis in the accessory lobe | Right cranial lobe (mild, lymphocytic bronchiolitis); right middle lobe (multifocal lobular atelectasis and interstitial pleocellular alveolitis, mild); left middle lobe (multilobular necrotizing, fibrinosuppurative, chronic  pneumonia); accessory lobe, multilobular atelectasis and diffuse pleocellular interstitial alveolitis with moderate multifocal ,  lymphonodular bronchitis,  no bacteria isolated from lung |
| 88 | Bilateral consolidation with focal pneumonia and pleuritis; 35% lung consolidated | Subacute necrotizing fibrinocellular lobar pneumonia and pleuritis, *Mannheimia haemolytica* isolated from lung  Moderate pleocellular tracheobronchitis  Bronchial lymph node: neutrophilic lymphadenitis |
| 99 | Unilateral focal lobar pneumonia and pleuritis, 20% lung consolidated | Subacute necrotizing fibrinocellular lobar pneumonia and pleuritis, *Mannheimia haemolytica* isolated |
| 113 | Unilateral focal lobar pneumonia with bilateral multifocal to coalescing atelectasis, 15% lung  consolidated | Subacute necrotizing fibrinocellular lobar pneumonia and pleuritis with diffuse atelectasis and mild BALT hyperplasia, *Mannheimia haemolytica* and *Mycoplasma bovis* isolated |
| 125 | Unilateral focal lobar pneumonia with mediastinal effusion, 30% lung consolidated. | Subacute necrotizing fibrinocellular lobar pneumonia and pleuritis, *Mannheimia haemolytica* and *Mycoplasma bovis*  *isolated* |

**B. *Pasteurella multocida***

| **Animal** | **Gross pathology** | **Histopathology/culture** |
| --- | --- | --- |
| 111 | Rare multifocal lobular atelectasis in the right cranial lung, 75% consolidation of the right middle lung, | Right middle lung: multifocal necrotizing, fibrinosuppurative, chronic pleuropneumonia with focally  extensive fibrosis and bronchiolitis obliterans; caudal right lung: severe lymphonodular, neutrophilic bronchopneumonia with bronchiolitis obliterans; tracheitis, mild to moderate, diffuse neutrophilic and lymphocytic. |
| 129 | No significant gross lesions | Right cranial lung lobe: focally extensive, chronic, lymphocytic bronchopneumonia with bronchiolitis obliterans.;Left cranial lobe: multifocal, lymphocytic bronchitis/bronchiolitis with multifocal pleocellular alveolitis; Right middle lobe: bronchitis, lymphocytic and neutrophilic with scant neutrophilic and lymphocytic exudate; Left  middle lobe; minimal multifocal, interstitial lymphocytic infiltrate. Mild diffuse pleocellular tracheitis. |
| 75 | Mild bilateral multilobular to coalescing consolidation involving approximately 10% of the lung,  *Mycoplasma bovis* isolated. | Mild, regional, bilateral, subacute lymphoid-lymphofollicular bronchitis/bronchiolitis with multifocal neutrophilic/histiocytic lobular bronchopneumonia.  *Mycoplasma bovis* isolated from lung |
| 123 | Minimal multilobular atelectasis/consolidation involving less than 1% of the lung, | Right middle lung lobe: focal necrotizing histiocytic pleocellular bronchopneumonia and lymphoid -lymphofollicular  bronchiolitis/bronchitis with chronic obliterating bronchiolitis.  b. Right apical, left apical and left middle lung lobes: mild lymphoid-lymphofollicular bronchitis/bronchiolitis.  *Mycoplasma bovis* and *M. canadens isolated from lung.* |
| 127 | Minimal lobular consolidation involving less than 1% of the lung, | Right apical lung lobe: focal chronic pleocellular bronchopneumonia and pleuritis with neutrophilic  lymphoid-lymphofollicular bronchiolitis/bronchitis  Mild, regional, bilateral, subacute lymphoid-lymphofollicular bronchitis/bronchiolitis with minimal neutrophilic  bronchopneumonia. *Mycoplasma bovis* isolated from lung |
| 135 | Mild multifocal bilateral consolidation involving 1-2% of the lung; | Right middle lung lobe: focal chronic pleocellular bronchopneumonia and pleuritis. Moderate, regional, bilateral, multifocal necrotizing histiocytic pleocellular bronchopneumonia with mild lymphoid to lymphofollicular bronchitis/bronchiolitis.  *Mycoplasma bovis* isolated from lung |

**C. *Mycoplasma bovis***

| **Animal** | **Gross pathology** | **Histopathology/culture** |
| --- | --- | --- |
| 42 | No significant gross lesions | Histologic change confined to major bronchus: minimal, lymphocytic, neutrophilic bronchitis  *Mycoplasma bovis* cultured from lung |
| 107 | Equivocal gross lesions; 0 % consolidation with slightly depressed discolored lobules in right apical lobe (30%), right middle lobe (60%) and right caudal lobe (20%) | Bilateral, multilobular, lymphocytic/lymphonodular bronchiolitis and mild diffuse lymphocytic and neutrophilic tracheitis  *Mycoplasma bovis* cultured from lung |
| 47 | Moderate bilateral consolidation involving 35% of the lung. | Bilateral neutrophilic bronchopneumonia with severe necrosuppurative obliterating bronchiolitis and  peribronchiolar/bronchial lymphoid hyperplasia  *Mycoplasma bovis* and *Mycoplasma bovirhinis* cultured from lung |
| 65 | Minimal lobular to multilobular atelectasis/consolidation involving approximately 3% of the lung | Mild lobular pleocellular bronchopneumonia with necrosuppurative obliterating bronchiolitis and  peribronchiolar/bronchial lymphoid hyperplasia.  *Mycoplasma bovis* cultured from lung |
| 115 | Mild multifocal lobular to multilobular atelectasis/consolidation involving approximately 5% of the lung. | Focal lobular pleocellular bronchopneumonia with necrosuppurative obliterating bronchiolitis and  peribronchiolar/bronchial lymphoid hyperplasia.  *Mycoplasma bovis* and *Mycoplasma. bovirhinis* cultured from lung. |

**D. *Histophilus somni***

| **Animal** | **Gross pathology** | **Histopathology/culture** |
| --- | --- | --- |
| 43 | Equivocal lesions were noted in the left middle lung lobe, consisting of slightly tan discolored lobules around the periphery of the ventral half of the lobe. The affected lobules are slightly collapsed with some interstitial fluid (edema) present. Otherwise the lung was unremarkable. | mild focal (left middle lung lobe) atelectasis with mild regional lymphoid  cuffing, bronchitis/bronchiolitis, No bacterial cultured from lung |
| 47 | Minimal changes were noted with some equivocal foci of patchy atelectasis in the mid portion of the right apical lobe. No other remarkable gross lesions were identified. | Regional, mild, multilobular,  fibrinoneutrophilic bronchopneumonia and mild BALT hyperplasia,  No bacteria cultured from lung |
