# Supplementary material for: Single Pathogen Challenge with Agents of the Bovine Respiratory Disease Complex
Source: PLoS One. 2015 Nov 16;10(11):e0142479. doi: 10.1371/journal.pone.0142479 (PMC4646450; doi:10.1371/journal.pone.0142479)
Supplement: S3 Table — (DOCX) [file pone.0142479.s003.docx]

**Table S3**. Bacterial isolations from lung and posterior pharyngeal swabs

**A. Viral Infected Animals**

| **BRSV Animal** | **Lung swab; bronchial swab** | **Posterior pharyngeal swab*** |
| --- | --- | --- |
| 18 | Negative for bacteria and Mycoplasma | *Pasteurella multocida*, mixed flora. *Mycoplasma bovirhinis* (day 7) |
| 33 | Lung: rare *P. multocida*, Bronchial swab: negative | *Pasteurella multocida*, mixed flora |
| 74 | Negative for bacteria and Mycoplasma | Mixed flora |
| 77 | Lung: Negative for bacteria and Mycoplasma, Bronchus: rare gram negative rod | Mixed flora |
| 92 | Negative for bacteria and Mycoplasma | Negative for bacteria and Mycoplasma |
| 116 | Negative for bacteria and Mycoplasma | Mixed flora |
| **BHV-2 (IBR) Animal** |  |  |
| 81 | Negative for bacteria and Mycoplasma | Pasteurella sp, Mannheimia sp, mixed flora, Mycoplasma sp |
| 138 | Negative for bacteria and Mycoplasma | Mixed flora; small number of Mannheimia sp |
| 59 | Lung: negative for bacteria and Mycoplasma; Bronchus: rare H. somni and *M. hemolytica* | D6: *Mannheimia haemolytica*  *Histophilus somni*, mixed flora |
| 70 | Negative for bacteria and Mycoplasma | D6: *Mannheimia haemolytica*,  mixed flora, Mycoplasma bovirhinis |
| 76 | Negative for bacteria and Mycoplasma | D6: *Mannheimia haemolytica*,  mixed flora, *Mycoplasma bovirhinis* |
| 100 | Negative for bacteria and Mycoplasma | D6: *Mannheimia haemolytica*,  *Pasteurella multocida*, mixed flora  *Histophilus somni, Mycoplasma bovirhinis* |
| **BVDV Animal** |  |  |
| 82 | Negative for bacteria and Mycoplasma | Pasteurella sp, Mannheimia sp, small number of mixed flora |
| 128 | Negative for bacteria and Mycoplasma | Small number of Pasteurella sp, Mannheimia sp |
| 86 | Negative for bacteria and Mycoplasma | *Mycoplasma hemolytica*, small #, *M. bovis, M. bovirhinis* |
| 98 | Negative for bacteria, Lung: Mycoplasma bovis | Negative for bacteria; *M. bovis* (D0), *M. bovirhinis* (D15) |
| 138 | Negative for bacteria, Lung: Mycoplasma bovis | *Mannheimia hemolytica* (D15); *Mycoplasma bovis* (D0,D15)), M. bovirhinis (D0) |
| 139 | Negative for bacteria, Lung: Mycoplasma bovis | Negative for bacteria; *M. bovis* (D15) |

**B. Bacterial Infected Animals**

| ***Mannheimia haemolytica* animal** | **Lung swab; bronchial swab** | **Posterior pharyngeal swab** |
| --- | --- | --- |
| 73 | Negative for bacteria and Mycoplasma | *Mannheimia haemolytica* ,Mixed flora |
| 75 | Negative for bacteria and Mycoplasma | Rare mixed flora |
| 88 | Lung: *Mannheimia haemolytica* Mod#  *Pasteurella multocida*, Bronchial: Mixed flora Sm#, *Mannheimia haemolytica*  *Pasteurella multocida* | *Mannheimia hemolytica, Mycoplasma bovirhinis*; |
| 99 | Lung: *Mannheimia haemolytica* Mod # Lung/Bronchial tissue pool: *M. bovirhinis* | Small number mixed flora, mycoplasma sp. |
| 113 | Lung and bronchial: *Mannheimia haemolytica* Lung/Bronchial tissue pool: *M. bovis* | *Mannheimia hemolytica*; rare mixed flora |
| 125 | Lung*: Mannheimia hemolytica,* Lung/Bronchial tissue pool: M. bovis | *Mannheimia hemolytica, Mycoplasma bovirhinis*; small number mixed flora |
| ***Pasteurella multocida* animal** |  |  |
| 111 | Lung: *Pasteurella multocida,* large # mixed flora; Bronchial swab negative | Pasteurella sp, Mannheimia sp, large number; *Mycoplasma bovirhinis* |
| 129 | No bacteria or mycoplasm isolated | Pasteurella sp, Mannheimia sp, large number |
| 75 | *M. bovis* isolated from lung/bronchial pool | Mixed flora |
| 123 | *M. bovis* isolated from lung/bronchial pool | Mixed flora, mycoplasma sp |
| 127 | *M. bovis* isolated from lung/bronchial pool | Mixed flora, mycoplasma sp |
| 135 | *M. bovis* isolated from lung/bronchial pool | Mixed flora, mycoplasma sp |
| ***Mycoplasma bovis* animal** |  |  |
| 42 | Negative for bacteria/ and Mycoplasma (M. bovis positive on lung/bronchus pool) | Pasteurella sp, Mannheimia sp, moderate number of mixed flora, *Mycoplasma bovis* |
| 107 |  | Pasteruella sp, Mannheimia sp.  Mycoplasma bovis |
| 47 | Negative for bacteria Lung: M. bovis, Bronchus: *M. bovirhinis* | Mycoplasma bovis (D15), *Histophilus somni, Mannheimia hemolytica* (small #), mixed flora, |
| 65 | Negative for bacteria M. bovis | *Mycoplasma bovis*, *Mannheimia hemolytica* (rare), mixed flora |
| 115 | Negative for bacteria Lung: M. bovis | *Mycoplasma bovirhinis* (D0), *Mycoplasma bovis* (D15), *Histophilus somni* (small # D15), *Mannheimia hemolytica* (small #), mixed flora, |
| ***Histophilus somni* animal** |  |  |
| 43 | Negative for bacteria; positive for *M.bovis* lung/bronchus pool | D0: *Mannheimia hemolytica* (moderate #); M. bovis; D7 *Pasteurella multocida* & mixed flora |
| 47 | Negative for bacteria | D0: *Pasteurella multocida, M. bovis*  D7: Pasteurella sp.; mixed flora |

**C. Mock-infected Control Animals**

| **Animal** | **Lung swab; bronchial swab** | **Posterial pharyngeal swab** |
| --- | --- | --- |
| Viral, 25 | Negative for bacteria/ mycoplasma | *Pasteurella multocida*, mixed flora |
| Viral, 58 | Lung: Negative for bacteria/ mycoplasma, Bronchus: *Mycoplasma bovirhinis* | *Mannheimia haemolytica* complex Sm#  Mixed flora Sm#, *Mycoplasma bovirhinis* |
| Viral, 101 | Negative for bacteria/ mycoplasma | Mannheimia haemolytica complex Rare  Mixed flora Rare |
| Bacterial, 70 | Lung: negative for bacteria/mycoplasma; Bronchus: *M. bovirhinis* | *Pasteurella multocida*, *Mannheimia hemolytica; Mycoplasma bovirhinis* |
| Bacterial, 71 | Negative for bacteria/ mycoplasma | *Mannheimia haemolytica* complex Rare  Mixed flora Rare, *Mycoplasma bovirhinis* |
| Bacterial, 102 | Negative for bacteria/ mycoplasma | Mixed flora, *Mycoplasma bovirhinis* |

*results include baseline before infection; when result changes during infection the day of isolation is indicated.
